# Hypoxia-induced apoptotic pathways and their contribution to fibrosis progression in wooden breast myopathy of fast-growing broilers

**DOI:** 10.1016/j.psj.2026.107805

**Published:** 2026-09-21

**Authors:** Shixiong Yan, Hongmei Shi, Muhammad Sarfaraz Iqbal, Gao Yang, Zhang Fei, Mikhlid H. Almutairi

**Affiliations:** a Yunnan Open University, Kunming, Yunnan, 650500, China; b State Key Laboratory of Microbial Metabolism, School of Life Sciences and Biotechnology, Shanghai Jiao Tong University, 800 Dongchuan Road, Minhang District, Shanghai, 200240, China; c College of Animal Science and Technology, Nanjing Agricultural University, No. 1 Weigang, Nanjing, Jiangsu, 210095, PR China; d Department of Animal Science, University of Arkansas, Fayetteville, AR, 72701, USA; e Department of Zoology, College of Science, King Saud University, P.O. Box 2455, Riyadh, 11451, Saudi Arabia

**Keywords:** Broiler, Fibrotic myopathy, HIF-1α, Apoptosis, Oxidative stress

## Abstract

Fibrotic breast myopathy, including the Wooden Breast (WB) condition, represents one of the most economically and welfare-significant challenges confronting modern commercial broiler production. Despite growing recognition of the syndrome, the cellular and molecular mechanisms linking chronic myofibre hypoxia to the progressive fibrotic pathology remain incompletely defined. This study employed a four-group experimental model using 192 male Ross 308 broilers subjected to chronic hypobaric hypoxia (simulated altitude 3,000 m; 13.0% O₂) with and without pharmacological pan-caspase inhibition via Z-VAD-FMK (2 mg/kg, three times weekly) over a 42-day production period, to characterise the hypoxia–apoptosis–fibrosis axis in the pectoralis major muscle. Chronic hypoxia induced progressive HIF-1α protein accumulation (4.8-fold by Day 42; p < 0.001), accompanied by marked elevation in apoptotic index (14.3% TUNEL-positive nuclei; p < 0.001), a strongly pro-apoptotic Bax/Bcl-2 ratio shift (8.2 vs. 1.0 in controls), and downstream activation of the intrinsic caspase cascade (cleaved caspase-9, caspase-3, and PARP). Concomitantly, hypoxic birds demonstrated severe oxidative stress (4.4-fold MDA elevation; depleted SOD, CAT, GPx, and GSH), robust NF-κB-mediated inflammation (IL-1β, IL-6, IL-8, TNF-α all elevated > 8-fold), and extensive extracellular matrix remodelling (hydroxyproline content 7.8 µg/mg; fibrotic area 31.4%; upregulation of *COL1A1, COL3A1*, TGF-β1, MMP-2, and MMP-9). The blockade of pan-caspases markedly decreased the severity of WB lesions, fibrosis (19.8%), apoptosis (6.1%), pro-inflammatory cytokines, and ECM turnover without affecting the upstream accumulation of HIF-1α, indicating that the executioner caspase activation was one of the key nodes of signal amplification during the development of the disease. A highly significant positive correlation between the expressions of HIF-1α, apoptotic index, hydroxyproline level, and NF-κB (r = 0.78-0.87; all P < 0.001) provided evidence for a mechanistic involvement of hypoxia-induced apoptosis in the pathogenesis of the disorder.

## Introduction

The production of broiler chickens is considered one of the most economically valuable parts of the world's animal husbandry, as they represent more than half of all meat protein produced in the world, and guarantee the existence of millions of farmers, processors, and workers within different geographical and socio-economic regions. The long process of genetic breeding for rapid growth, large breast muscle mass, and high feed efficiency in commercial broilers resulted in broiler chickens that were beyond the wildest imagination of previous generations of poultry scientists. Commercial broilers currently reach their slaughter weights in less than 35 days, a physiological feat that has helped bring down the price of poultry protein in comparison to other sources of animal proteins and ensured affordable access to protein in diets for people from all economic backgrounds (Petracci et al., 2019; Sihvo et al., 2014). Despite the presence of pathological similarities between fibrotic breast myopathy and other forms of myopathies, the distinguishing characteristic in the case of fibrotic breast myopathy is the presence of an attribute whereby myofibres are replaced with collagenous connective tissues, which signify the healing process resulting from continuous or repetitive injury of the muscle caused by metabolic pressure in broiler chickens (Velleman and Clark, 2015; Papah et al., 2017). Prevalence rates of white striations and wooden breast in processing samples vary between 5% and more than 50%, according to the applied criteria of scoring and the genetic line and management conditions investigated, with the highest prevalence rates being recorded for the heaviest and fastest-growing lines (Kuttappan et al., 2013; Tijare et al., 2016). The yearly financial losses incurred from these myopathies have been calculated to be worth hundreds of millions of dollars worldwide because of a combination of factors such as low yield of fillets, product downgrading, higher processing losses, limited export opportunities, and consumer rejection. In addition to their effects on the economy, however, breast muscle myopathies have serious implications concerning animal welfare, since the processes leading to conditions like fibrotic breast myopathy, including ischaemia, hypoxia, oxidative stress, inflammation, necrosis of myofibers, and apoptosis of cells, may likely be painful to the animals suffering from the disease and have therefore become an important aspect considered in welfare regulation legislation in several countries (Tijare et al., 2016; Soglia et al., 2016). The cellular response to hypoxia in vertebrate cells is largely controlled by the hypoxia-inducible transcription factor system, with HIF-1α being one of the best studied and most physiologically relevant components of the pathway regulating adaptive changes under hypoxia (Semenza, 2012). In the presence of normal levels of oxygen, HIF-1α protein is quickly hydroxylated at conserved proline amino acids in its oxygen-sensitive degradation domain by prolyl-hydroxylase domain (PHD) proteins and thus tagged for ubiquitin-mediated proteolysis by von Hippel-Lindau tumor suppressor protein (VHL) with a half-life of just a few minutes (Kaelin and Ratcliffe, 2008). As long as the cellular oxygen concentration remains lower than the cut-off level for PHD functioning, HIF-1α is no longer hydroxylated, and it can therefore be accumulated and transported to the nucleus to bind to its constant co-activator HIF-1β (ARNT) in order to form an active complex capable of activating the expression of hundreds of genes coding for functions related to angiogenesis, glycolysis, erythropoiesis, cell survival, and apoptosis (Semenza, 2012; Keith et al., 2012). The present study was designed to address these knowledge gaps through a comprehensive characterization of the molecular pathways linking chronic tissue hypoxia to apoptotic myofibre death in the pectoralis major muscle of commercial broilers exhibiting macroscopic and histopathological features of fibrotic breast myopathy. To achieve a controlled and reproducible hypoxic insult, we employed a hypobaric hypoxia model simulating an altitude of 3,000 m (13.0% O₂). This model was selected based on prior validation studies (Sihvo et al., 2018; Meloche et al., 2018) as a reproducible means to induce chronic systemic hypoxia and WB-like lesions under controlled experimental conditions, independent of regional farming geography. While systemic hypoxia differs etiologically from the local microvascular insufficiency hypothesised to drive natural WB, it provides a standardized platform for mechanistic dissection of the hypoxia–apoptosis–fibrosis axis.

## Materials and methods

### Ethical approval and animal care

All animal procedures were approved by Animal Care and Use Committee (SYXK-2025-057) in accordance with the guidelines of the National Research Council Guide for the Care and Use of Laboratory Animals (Bayne et al., 2026) and the European Union Directive 2010/63/EU on the protection of animals used for scientific purposes. The study was conducted at a biosafety level-2 accredited poultry research facility. Birds were monitored twice daily for welfare indicators including feed and water intake, mobility, respiratory distress, and mortality, and were humanely euthanised by cervical dislocation at predetermined endpoints.

### Experimental animals, design, and housing

A total of 192 male Ross 308 broiler chicks (Gallus gallus domesticus) sourced from a single commercial hatchery were allocated into four experimental groups (n = 48 per group) using a completely randomised design: (i) Control (normoxia + saline vehicle), (ii) Hypoxia (chronic hypobaric hypoxia + saline vehicle), (iii) Apoptosis-Inhibitor (normoxia + pan-caspase inhibitor Z-VAD-FMK), and (iv) Combined (chronic hypobaric hypoxia + Z-VAD-FMK). Group allocation was stratified by initial body weight to achieve comparable mean baseline weights (43.3 ± 2.0 g) across groups (see Supplementary Table S1). Only male birds were used to eliminate confounding from sexual dimorphism in growth rate and breast muscle development.

Hypoxia and Combined groups were housed in purpose-built hypobaric chambers (0.5 m³ capacity each, internal floor area 0.60 m²; Altitrainer 200, SMTEC, Switzerland) maintained at a simulated altitude of 3,000 m above sea level (barometric pressure 70.1 kPa, O₂ partial pressure equivalent to 13.0 ± 0.3%). To avoid extreme stocking density, birds were distributed across four chambers per group (12 birds per chamber), providing an initial floor area of 0.05 m² per bird. Control and Apoptosis-Inhibitor groups were housed in floor pens with matched floor area per bird (0.05 m² per bird). Final stocking density at Day 42 was approximately 48 kg/m² in hypobaric chambers (Hypoxia group) and 58 kg/m² in normoxic floor pens (Control group). All birds were maintained under a 23 L:1 D photoperiod, fed a commercial corn–soybean meal basal diet (nutritional composition provided in Supplementary Table S2), and given ad libitum access to water throughout the 42-day experimental period. No sham chamber control (normobaric conditions within an identical chamber enclosure) was employed.

### Pharmacological inhibition of apoptosis

The broad-spectrum pan-caspase inhibitor Z-Val-Ala-Asp(OMe)-fluoromethyl ketone (Z-VAD-FMK; Selleckchem, Houston, TX, USA; Catalogue No. S7023) was dissolved in dimethyl sulfoxide (DMSO; Sigma-Aldrich, St. Louis, MO, USA) and diluted in sterile physiological saline to a final DMSO concentration of ≤ 1% (v/v). Birds in the Apoptosis-Inhibitor and Combined groups received Z-VAD-FMK at a dose of 2 mg/kg body weight administered via intramuscular injection into the pectoralis minor muscle three times per week, commencing on Day 7 and continuing until the final sampling point (Day 42), as adapted from the protocol of Oltvai et al. (1993). The pectoralis minor was selected as the injection site to avoid direct mechanical and chemical trauma to the target tissue (pectoralis major). Z-VAD-FMK is a cell-permeable compound expected to distribute systemically following intramuscular absorption; however, no pharmacokinetic validation (e.g., plasma or pectoralis major tissue concentrations) was performed in the present study to confirm therapeutic levels in the target muscle. Birds were monitored daily for injection-site reactions (swelling, erythema, induration); no overt local adverse reactions were observed. Control and Hypoxia groups received equivalent volumes of the vehicle (1% DMSO in saline) on the same schedule.

### Tissue collection and sample processing

Twelve birds per group were euthanised by cervical dislocation at Days 14, 28, and 42 post-hatch. The entire pectoralis major (PM) muscle was excised immediately after euthanasia, grossly inspected, and scored for WB lesion severity using the four-point standardised scale (0–3) described by Kuttappan et al. (2013) (full scoring criteria in Supplementary Table S5). Tissue samples from the cranial third of the left PM muscle were collected for the following purposes: (i) snap-frozen in liquid nitrogen and stored at −80°C for molecular analyses; (ii) fixed in 10% neutral-buffered formalin (NBF, pH 7.4) for 24 h and embedded in paraffin (FFPE) for histology and immunohistochemistry; (iii) embedded in optimal cutting temperature (OCT) compound and frozen for TUNEL assay and immunofluorescence; and (iv) placed in RNA-later stabilisation solution (ThermoFisher Scientific, Waltham, MA, USA) for RNA extraction. All tissue handling was performed on ice.

### Histopathology and lesion scoring

Formalin-fixed, paraffin-embedded tissue blocks were sectioned at 5 µm thickness using a rotary microtome (Leica RM2255, Leica Biosystems, Germany). Serial sections were stained with Haematoxylin and Eosin (H&E) for general morphology, Masson's Trichrome (MT) for collagen/fibrosis assessment, and Periodic Acid-Schiff (PAS) for polysaccharide deposits. Stained slides were digitised using a Hamamatsu NanoZoomer S360 whole-slide imaging system and evaluated independently by two board-certified veterinary pathologists blinded to treatment group. Quantitative morphometry was performed on H&E-stained sections using QuPath v0.4.3 software (Bankhead et al., 2017). The percentage area of interstitial fibrosis was determined on MT-stained sections by colour deconvolution and thresholding of the blue channel (collagen) relative to total tissue area in five randomly selected fields per section (× 100 magnification), with a minimum measurement area of 1.0 mm² per field.

### RNA extraction and reverse transcription-quantitative PCR (RT-qPCR)

Total RNA was extracted from 50 mg of snap-frozen PM muscle using TRIzol Reagent (ThermoFisher Scientific) according to the manufacturer's instructions. RNA purity and concentration were assessed spectrophotometrically (NanoDrop One, ThermoFisher Scientific); samples with A260/280 ratio < 1.8 or A260/230 ratio < 1.7 were excluded. RNA integrity was confirmed by automated capillary electrophoresis (Agilent Bioanalyzer 2100); all samples had RNA Integrity Number (RIN) ≥ 7.5. cDNA was synthesised from 1 µg total RNA using the High-Capacity cDNA Reverse Transcription Kit (Applied Biosystems, Foster City, CA, USA) in 20 µL reactions according to the manufacturer's protocol.

Gene-specific primers for Gallus gallus target genes (*HIF1A, VEGFA, BAX, BCL2, CASP3, COL1A1, COL3A1, MMP2, MMP9, TGFB1*) and reference genes (*GAPDH, RPL19*) were designed using Primer3 v4.1.0 and verified for specificity against the Gallus gallus genome (GalGal6) using NCBI Primer-BLAST (Ye et al., 2012). Primer sequences, amplicon sizes, and PCR efficiencies are provided in Supplementary Table S3. RT-qPCR was performed in triplicate 10 µL reactions on a CFX384 Touch Real-Time PCR Detection System (Bio-Rad, Hercules, CA, USA) using iTaq Universal SYBR Green Supermix (Bio-Rad). Thermal cycling conditions consisted of initial denaturation at 95°C for 3 min, followed by 40 cycles of 95°C for 10 s and 60°C for 30 s, with melt curve analysis from 65 to 95°C. Relative gene expression was calculated using the 2^(−ΔΔCt) method (Livak and Schmittgen, 2001) with *GAPDH* and *RPL19* as co-reference genes; reference gene stability was confirmed using geNorm (Vandesompele et al., 2002).

### Protein extraction and western blotting

Snap-frozen PM muscle (50 mg) was homogenised in RIPA lysis buffer (150 mM NaCl, 1% Triton X-100, 0.5% sodium deoxycholate, 0.1% SDS, 50 mM Tris-HCl, pH 8.0) supplemented with protease and phosphatase inhibitor cocktails (Roche, Basel, Switzerland) using a TissueLyser LT (Qiagen, Hilden, Germany) at 50 Hz for 2 × 2 min. Lysates were centrifuged at 14,000 × g for 15 min at 4°C, and the protein-containing supernatant was collected. Total protein concentration was determined by bicinchoninic acid (BCA) assay (Pierce BCA Protein Assay Kit, ThermoFisher Scientific).

Proteins (20 µg/lane) were resolved by 10—12% SDS-PAGE and transferred to 0.2 µm polyvinylidene difluoride (PVDF) membranes (Bio-Rad) by wet transfer at 100 V for 90 min. Membranes were blocked in 5% non-fat dried milk in Tris-buffered saline with 0.1% Tween-20 (TBST) for 1 h at room temperature, then incubated with primary antibodies overnight at 4°C with gentle agitation. Details of all primary and secondary antibodies, including dilutions and suppliers, are provided in Supplementary Table S4. HRP-conjugated secondary antibodies (1:5,000; Cell Signaling Technology, Danvers, MA, USA) were applied for 1 h at room temperature, and bands were detected by enhanced chemiluminescence (ECL Select, Cytiva, Marlborough, MA, USA) on a ChemiDoc MP Imaging System (Bio-Rad). Densitometric quantification of band intensities was performed using ImageJ v1.53 (Schneider et al., 2012), with all values normalised to the β-actin loading control.

### Immunohistochemistry (IHC)

IHC was performed on 5 µm FFPE sections using the avidin-biotin-peroxidase complex (ABC) method (Vectastain Elite ABC—HRP Kit, Vector Laboratories, Newark, CA, USA). Briefly, sections were deparaffinised, rehydrated, and subjected to heat-induced epitope retrieval in 10 mM citrate buffer (pH 6.0) at 100°C for 20 min. Endogenous peroxidase activity was quenched with 3% H2O2 in methanol for 10 min, and non-specific binding was blocked with 5% normal goat serum (Vector Laboratories) for 30 min at room temperature. Primary antibodies against HIF-1α, VEGF-A, cleaved Caspase-3, Bax, and MMP-2 (dilutions in Supplementary Table S4) were applied overnight at 4°C. Biotinylated secondary antibodies were applied for 30 min, followed by the ABC reagent for 30 min. Immunoreactivity was visualised using 3,3′-diaminobenzidine (DAB) as the chromogen, and sections were counterstained with Mayer's haematoxylin. Negative controls, omitting the primary antibody, were included in all runs. IHC staining was semi-quantitatively scored (0–3+ scale) by two blinded pathologists using Allred-modified criteria.

### TUNEL assay for apoptosis detection

Terminal deoxynucleotidyl transferase dUTP nick-end labelling (TUNEL) assay was performed on 7 µm cryosections using the In Situ Cell Death Detection Kit (Fluorescein, Roche) according to the manufacturer's instructions. Sections were post-fixed in 4% paraformaldehyde for 20 min, permeabilised with 0.1% Triton X-100 and 0.1% sodium citrate for 2 min on ice, and incubated with the TUNEL reaction mixture at 37°C for 60 min in a humidified chamber. Nuclei were counterstained with 4′,6-diamidino-2-phenylindole (DAPI; 1 µg/mL). Slides were imaged on a Leica DMi8 fluorescent microscope with appropriate filter sets (FITC and DAPI channels). The apoptotic index (AI) was calculated as the percentage of TUNEL-positive nuclei per total number of DAPI-positive nuclei in a minimum of five randomly selected fields per section (× 200 magnification), with at least 1,000 nuclei counted per sample.

### Oxidative stress biomarker assays

Approximately 100 mg of snap-frozen muscle tissue was homogenised in 50 mM phosphate buffer (pH 7.4) at 4°C (10% w/v), centrifuged at 10,000 × g for 15 min, and the resulting supernatant used for enzymatic and spectrophotometric assays. All assay kits were sourced from Cayman Chemical (Ann Arbor, MI, USA) unless otherwise stated. Malondialdehyde (MDA) was quantified as a thiobarbituric acid-reactive substance (TBARS) using the TBARS Assay Kit (Item No. 10009055). Protein carbonyl content was determined by 2,4-dinitrophenylhydrazine (DNPH) derivatisation using the Protein Carbonyl Colorimetric Assay Kit (Item No. 10005020). Superoxide dismutase (SOD) activity was measured by the WST-1 method (SOD Assay Kit, Item No. 706002). Catalase (CAT) activity was determined by the colorimetric quantification of H2O2 consumption (Catalase Assay Kit, Item No. 707002). Glutathione peroxidase (GPx) activity was measured by the coupled NADPH-dependent assay (GPx Assay Kit, Item No. 703102). Total glutathione (GSH + GSSG) was quantified fluorometrically using the GSH/GSSG-Glo Assay (Promega, Madison, WI, USA). All values were normalised to total protein concentration determined by the Bradford method.

### Cytokine quantification

Muscle homogenates were prepared in cold Cell Extraction Buffer (ThermoFisher Scientific) supplemented with protease inhibitors. Cytokine concentrations of interleukin-1β (IL-1β), IL-6, IL-8, tumour necrosis factor-α (TNF-α), and transforming growth factor-β1 (TGF-β1) were simultaneously quantified using a validated chicken-specific multiplex bead-based ELISA panel (LEGENDplex Chicken Inflammation Panel, BioLegend, San Diego, CA, USA) on a FACSCanto II flow cytometer (BD Biosciences, San Jose, CA, USA) with LEGENDplex v8.0 software for data analysis. Results were expressed as picograms per milligram total protein, normalised to the BCA assay.

### Hydroxyproline assay for collagen quantification

Total collagen content in PM muscle was estimated by tissue hydroxyproline content using the Hydroxyproline Assay Kit (Sigma-Aldrich, MAK008) according to the manufacturer's protocol. Briefly, 10 mg of lyophilised muscle tissue was hydrolysed in 6 N HCl at 120°C for 3 h, evaporated to dryness, resuspended, and reacted with chloramine-T and Ehrlich's reagent. Absorbance was measured at 560 nm on a SpectraMax iD5 microplate reader (Molecular Devices, San Jose, CA, USA). Hydroxyproline concentrations were calculated from a trans-4‑hydroxy-L-proline standard curve (0–5 µg/mL) and expressed as micrograms per milligram wet tissue weight.

### Gelatin zymography for MMP activity

Gelatinolytic activities of MMP-2 and MMP-9 were assessed in muscle protein extracts (50 µg protein/lane) by gelatin zymography. Samples were resolved under non-reducing conditions on 10% SDS-PAGE gels co-polymerised with 0.1% (w/v) gelatin (Sigma-Aldrich). Following electrophoresis, gels were washed twice in 2.5% Triton X-100 for 30 min to remove SDS, then incubated in developing buffer (50 mM Tris-HCl, pH 7.5, 5 mM CaCl2, 0.2 M NaCl, 0.02% NaN3) at 37°C for 48 h. Gels were stained with 0.5% Coomassie Brilliant Blue R-250 and destained in 30% methanol/10% acetic acid. Clear bands of gelatinolytic activity against a blue background were visualised and densitometrically quantified using ImageJ v1.53, with band positions confirmed against purified MMP-2 and MMP-9 standards (R&D Systems, Minneapolis, MN, USA).

### NF-κB nuclear translocation assay

Nuclear and cytoplasmic protein fractions were isolated from PM muscle using the Nuclear Extract Kit (Active Motif, Carlsbad, CA, USA) following the manufacturer's instructions. NF-κB p65 content in nuclear fractions was assessed by both Western blotting (as described in Section 2.7) and electrophoretic mobility shift assay (EMSA) using the LightShift Chemiluminescent EMSA Kit (ThermoFisher Scientific) with a biotin-labelled NF-κB consensus oligonucleotide (5′-AGTTGAGGGGACTTTCCCAGGC-3′). Lamin B1 and *GAPDH* were used as nuclear and cytoplasmic loading controls, respectively.

### Statistical analysis

All data were tested for normality using the Shapiro-Wilk test and for homogeneity of variances using Levene's test prior to parametric analyses. Continuous variables were analysed primarily by one-way analysis of variance (ANOVA) performed independently at each sampling time point (Days 14, 28 and 42), followed by Tukey’s Honestly Significant Difference (HSD) post-hoc test. This approach treats each time point as a separate completely randomised experiment and directly addresses group differences at the biologically relevant stages of lesion development.In addition, a two-way ANOVA with Group and Time as fixed factors was performed to evaluate overall temporal patterns and possible Group × Time interactions. While the interaction term is reported for completeness, interpretation of the results focuses on the simple main effects at each time point, which are most relevant to the hypoxia–apoptosis–fibrosis causal chain under investigation. Non-parametric data (WB lesion scores, IHC scores) were analysed by Kruskal–Wallis test with Dunn’s post-hoc correction at each time point.Pearson correlation analyses were restricted to Days 28 and 42 (n = 72) as described previously. Statistical significance was set at α = 0.05. Effect sizes are reported as partial eta-squared (η²p). Full ANOVA tables, including one-way analyses at each time point, are provided in Supplementary Table S8. For all tests, statistical significance was set at α = 0.05. Effect sizes are reported as partial eta-squared (η²p) for ANOVA comparisons. All statistical analyses were conducted in GraphPad Prism v10.1 (GraphPad Software, Boston, MA, USA) and SPSS v29.0 (IBM Corp., Armonk, NY, USA). A complete statistical summary including F-statistics, degrees of freedom, p-values, and η²p values for all primary outcomes is provided in Supplementary Table S8.

## Results

### Mortality, body weight, and gross pathology

Overall mortality rate from day 1 until day 42 was 3.1%, 8.3%, 4.2%, and 7.1% in Control, Hypoxia, Apoptosis-Inhibitor, and Combined groups, respectively, with a significant increase in mortality rate for hypoxia-exposed groups when compared with normoxic controls (χ² = 12.4, p = 0.006). Mortality in all hypoxia exposed groups occurred only between days 14 and 28 and was caused by ascites and right ventricular failure due to hypoxia-induced pulmonary hypertension syndrome. Birds that died spontaneously were not included in downstream tissue analyses because carcasses were frequently autolytic or compromised by severe systemic oedema, precluding reliable pectoralis major dissection and WB lesion scoring. Consequently, the Day 42 Hypoxia cohort represents hypoxia-tolerant survivors, and reported pathology metrics may underestimate the true severity in the full exposed population. Body weight gain was significantly lower in the hypoxia-exposed animals starting on day 28 (day 42: 2,418 ± 87 g vs. 2,891 ± 94 g in Control; p < 0.001). Notably, lower body weight is typically associated with reduced WB severity in commercial broilers; therefore, the markedly elevated pathology observed in the Hypoxia group occurred despite, not because of, this growth depression. Z-VAD-FMK treatment partially attenuated hypoxia-induced growth depression (Combined group Day 42: 2,634 ± 79 g; p < 0.05 vs. Hypoxia group; Fig. 1). WB lesion prevalence increased markedly with age in hypoxia-exposed birds. At Day 42, the Hypoxia group exhibited significantly higher WB lesion scores (median score 2.5, IQR 2.0–3.0) compared with the Control group (median score 0, IQR 0–0.5; Kruskal-Wallis p < 0.001). The Apoptosis-Inhibitor group under normoxia showed minimal lesion prevalence comparable to the Control (median score 0.5, IQR 0–1.0), indicating that repeated pectoralis minor injection of Z-VAD-FMK did not induce WB-like pathology in the pectoralis major. Z-VAD-FMK treatment of hypoxic birds (Combined group) significantly reduced lesion scores at Day 42 (median score 1.5, IQR 1.0–2.0; p < 0.01 vs. Hypoxia), confirming a functional role for caspase-dependent apoptosis in WB myopathy progression (Fig. 2A–B).

**Fig. 1:**
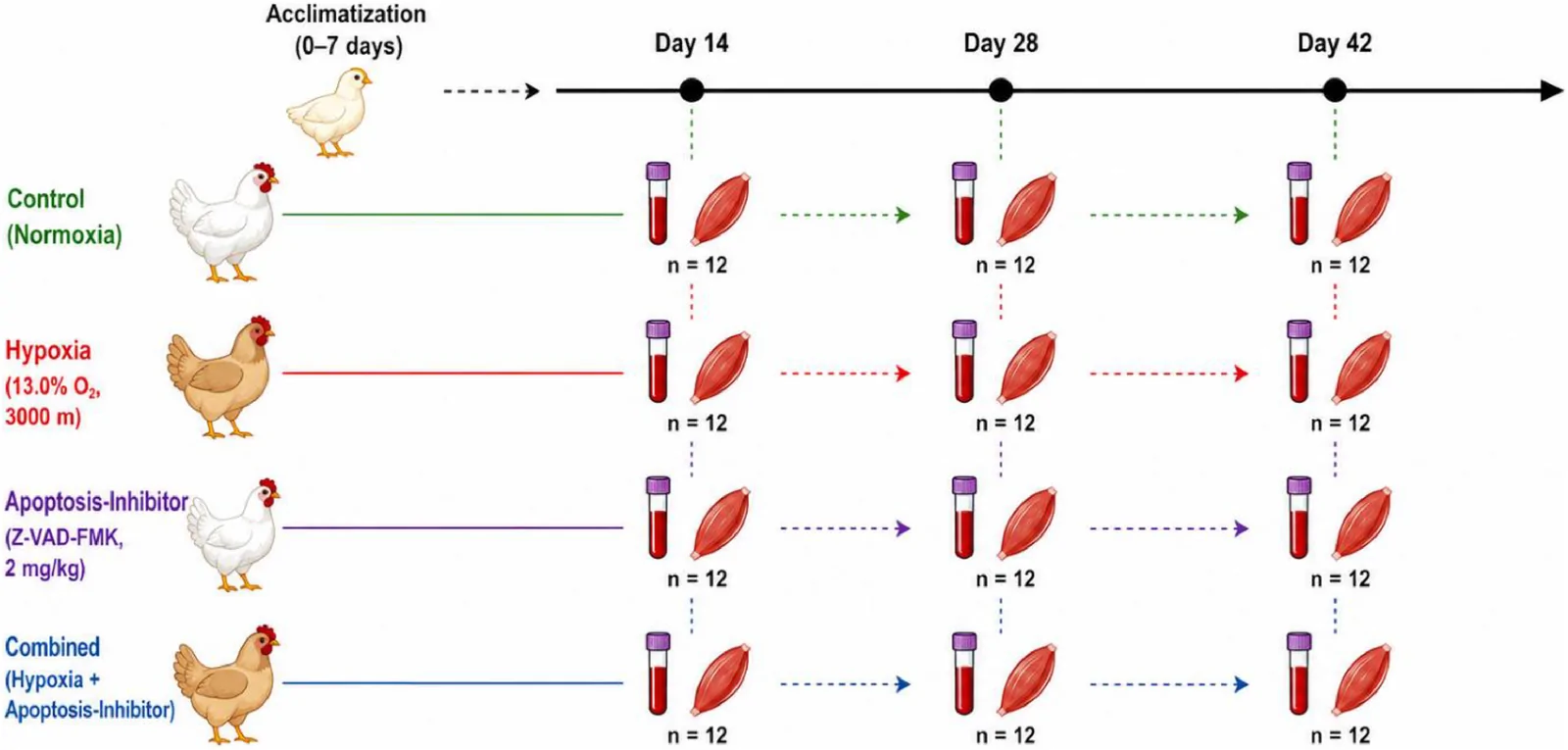
Experimental Design and Animal Grouping Timeline. Schematic overview of the experimental design showing four treatment groups (Control, Hypoxia, Apoptosis-Inhibitor, and Combined), sampling timepoints at Days 14, 28, and 42, and key assays performed at each interval. Birds are depicted with colour-coded group identification. n = 12 birds per group per timepoint.

**Fig. 2:**
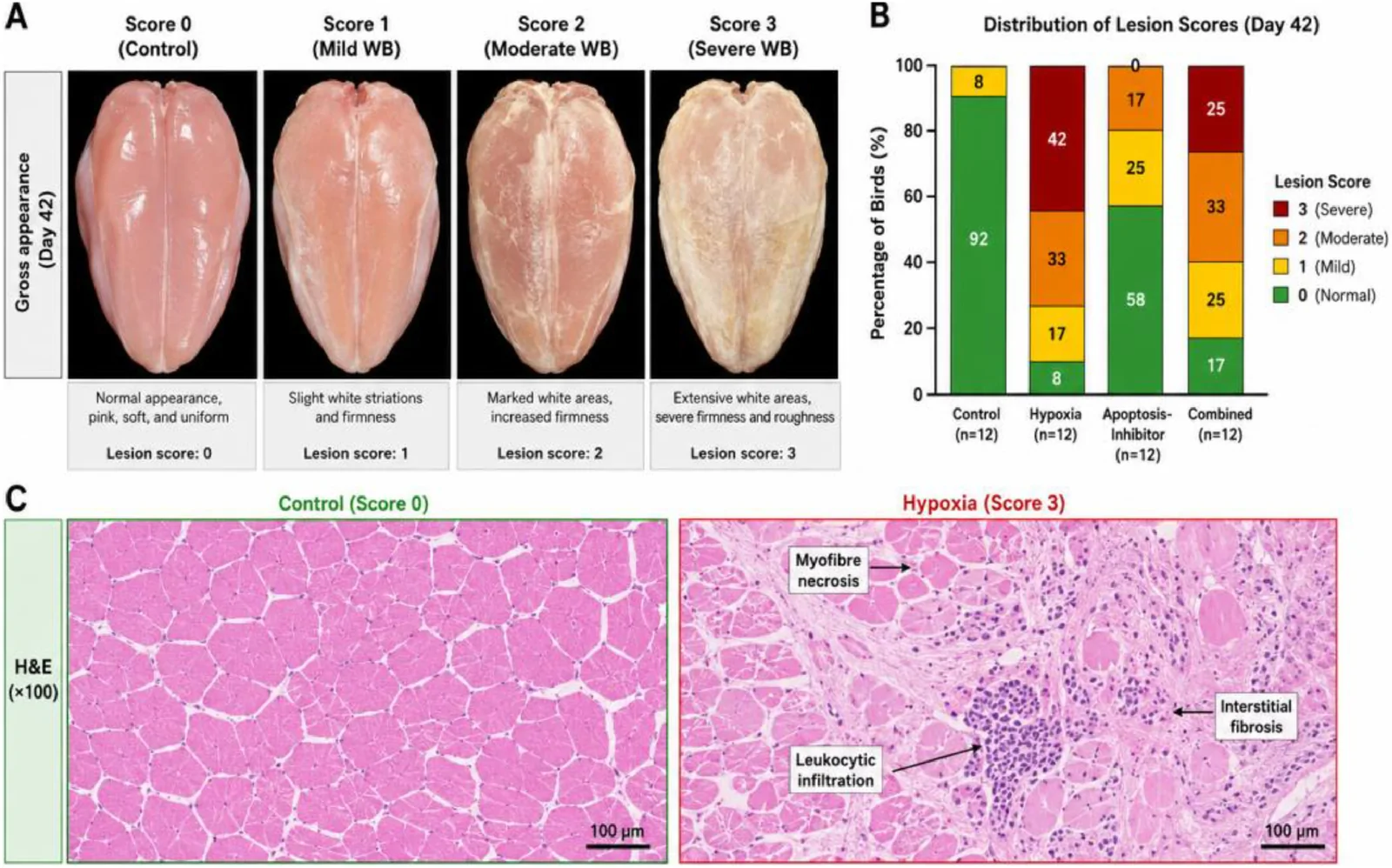
Gross and Histopathological Lesion Scoring of Broiler Breast Muscle. (A) Representative photographs of pectoralis major muscle from Control (score 0), mild Wooden Breast (score 1), moderate WB (score 2), and severe WB (score 3) broilers at Day 42. (B) Stacked bar chart showing distribution of lesion scores across groups. (C) Haematoxylin and eosin (H&E)-stained transverse sections (× 100) showing normal fascicular architecture (Control) versus myofibre necrosis, interstitial fibrosis, and leukocytic infiltration (Hypoxia group). Scale bar = 100 µm.

### Histopathological assessment

H&E examination of pectoralis major sections revealed progressive and time-dependent histopathological lesions in the Hypoxia group (Fig. 2C). At Day 14, mild vacuolar degeneration of isolated myofibres was observed in hypoxic birds, with a mean lesion area fraction of 4.8 ± 1.2% compared with 0.4 ± 0.2% in Controls (p < 0.05). By Day 28, multifocal myofibre necrosis with phagocytosis, interstitial oedema, and infiltration of macrophages and heterophils were evident in the Hypoxia group (lesion area fraction 18.7 ± 2.3%). At Day 42, the Hypoxia group demonstrated extensive myofibre replacement by fibrotic connective tissue, with Masson's Trichrome confirming a mean fibrotic area of 31.4 ± 3.1% of total section area. In contrast, the Control group exhibited preserved fascicular architecture throughout with minimal (<2%) fibrotic area at Day 42.

Z-VAD-FMK treatment significantly attenuated histopathological severity in the Combined group, with Day 42 lesion area fractions of 18.2 ± 2.6% (p < 0.01 vs. Hypoxia group) and fibrotic area of 19.8 ± 2.4% (p < 0.01 vs. Hypoxia). Muscle fibre cross-sectional area (CSA) measured by morphometry was significantly reduced in the Hypoxia group at Days 28 and 42 (Day 42: 4,012 ± 296 µm² vs. 6,104 ± 418 µm² in Control; p < 0.001), consistent with myofibre atrophy secondary to hypoxic injury (Supplementary Table S7). A significant increase in the proportion of small-diameter (atrophied) fibres was observed in the Hypoxia group, accompanied by a shift toward Type IIb fibre predominance at Day 42 (68.4 ± 4.2% vs. 55.9 ± 3.6% in Control; p < 0.001).

### Hypoxia-inducible factor-1α (HIF-1α) expression

Western blot analysis demonstrated a significant progressive elevation of HIF-1α protein in the Hypoxia group across all timepoints, reaching a 4.8 ± 0.4-fold increase over Day-14 Control at Day 42 (p < 0.001; Fig. 3A-B). The increase in HIF-1α was detectable from Day 14 (1.9 ± 0.2-fold; p < 0.05 vs. Control) and amplified markedly by Day 28 (3.1 ± 0.3-fold; p < 0.001). During normoxia (Control and Apoptosis-Inhibitor groups), HIF-1α protein expression maintained basal level values due to prolyl hydroxylase-mediated degradation of HIF-1α protein (Maxwell et al., 1999).

**Fig. 3:**
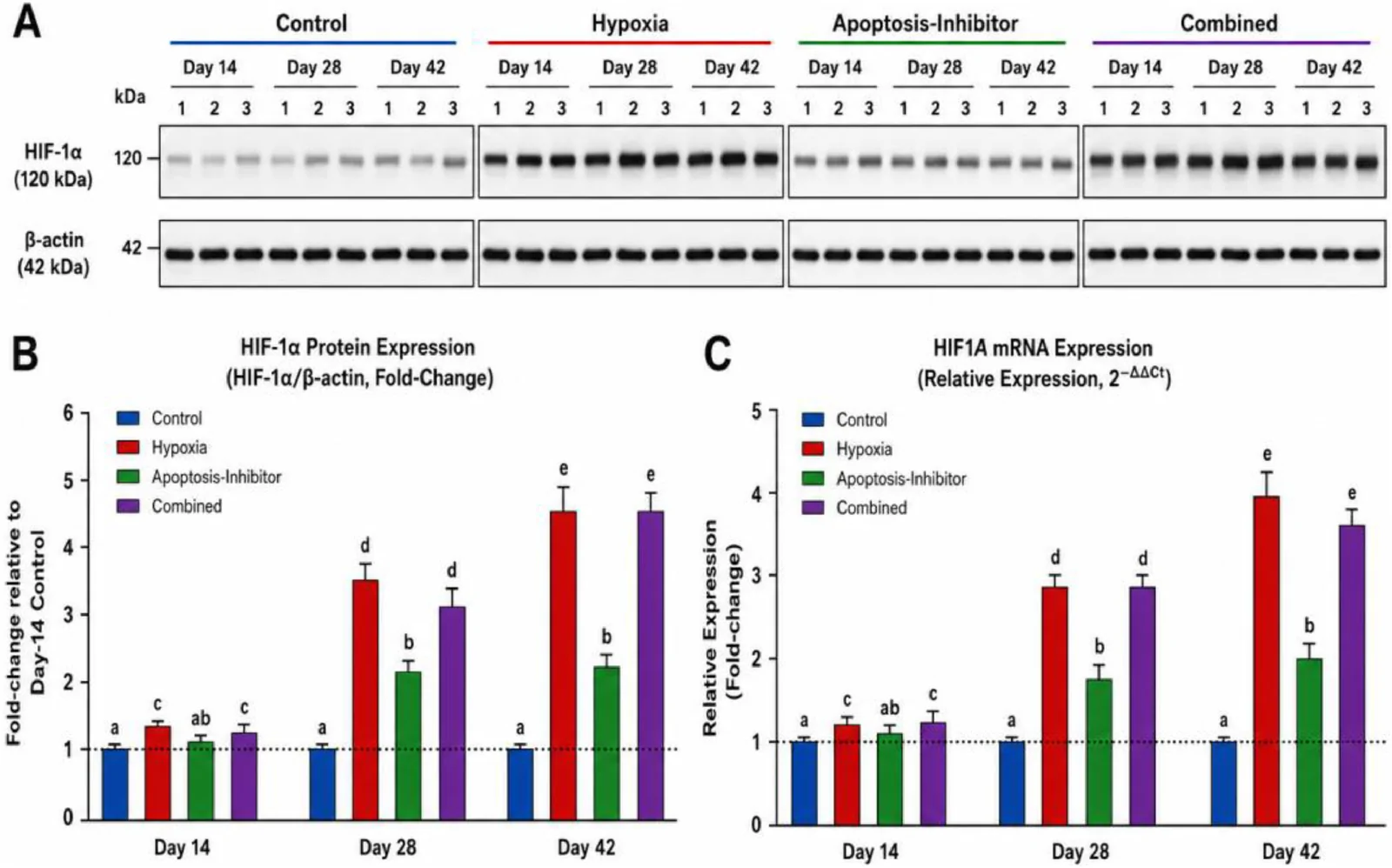
Hypoxia-Inducible Factor-1α (HIF-1α) Protein and mRNA Expression Profiles. (A) Representative Western blot images for HIF-1α and β-actin loading control across all groups at Days 14, 28, and 42. (B) Densitometric quantification of HIF-1α/β-actin ratios expressed as fold-change relative to Day-14 Control (mean ± SEM, n = 6). (C) RT-qPCR relative quantification of HIF1A mRNA (2^−ΔΔCt method, normalised to GAPDH and RPL19).

At the same time, an increased level of *HIF1A* mRNA in the Hypoxia group (by RT-qPCR; Fig. 3C: 3.7 ± 0.3-fold on Day 42 vs. Control group; p < 0.001) indicated transcriptional activation. Treatment with Z-VAD-FMK in the Combined group resulted in no statistically significant changes in HIF-1α expression compared with the Hypoxia group (p > 0.05), thus suggesting that caspases' inhibition did not affect this parameter. *VEGFA* mRNA level correlated positively with the changes in *HIF1A* in the Hypoxia group (Day 42: 2.9 ± 0.2-fold increase; p < 0.001). This result is in agreement with data on the involvement of HIF-1α protein in the transcriptional activation of the VEGF promoter (Semenza, 2012). Immunohistochemistry confirmed the strong staining for HIF-1α in the nuclei of myofibers and interstitial cells of the Hypoxia group with the semi-quantitative score value 2.8 ± 0.2 vs. 0, Fig. 4).

**Fig. 4:**
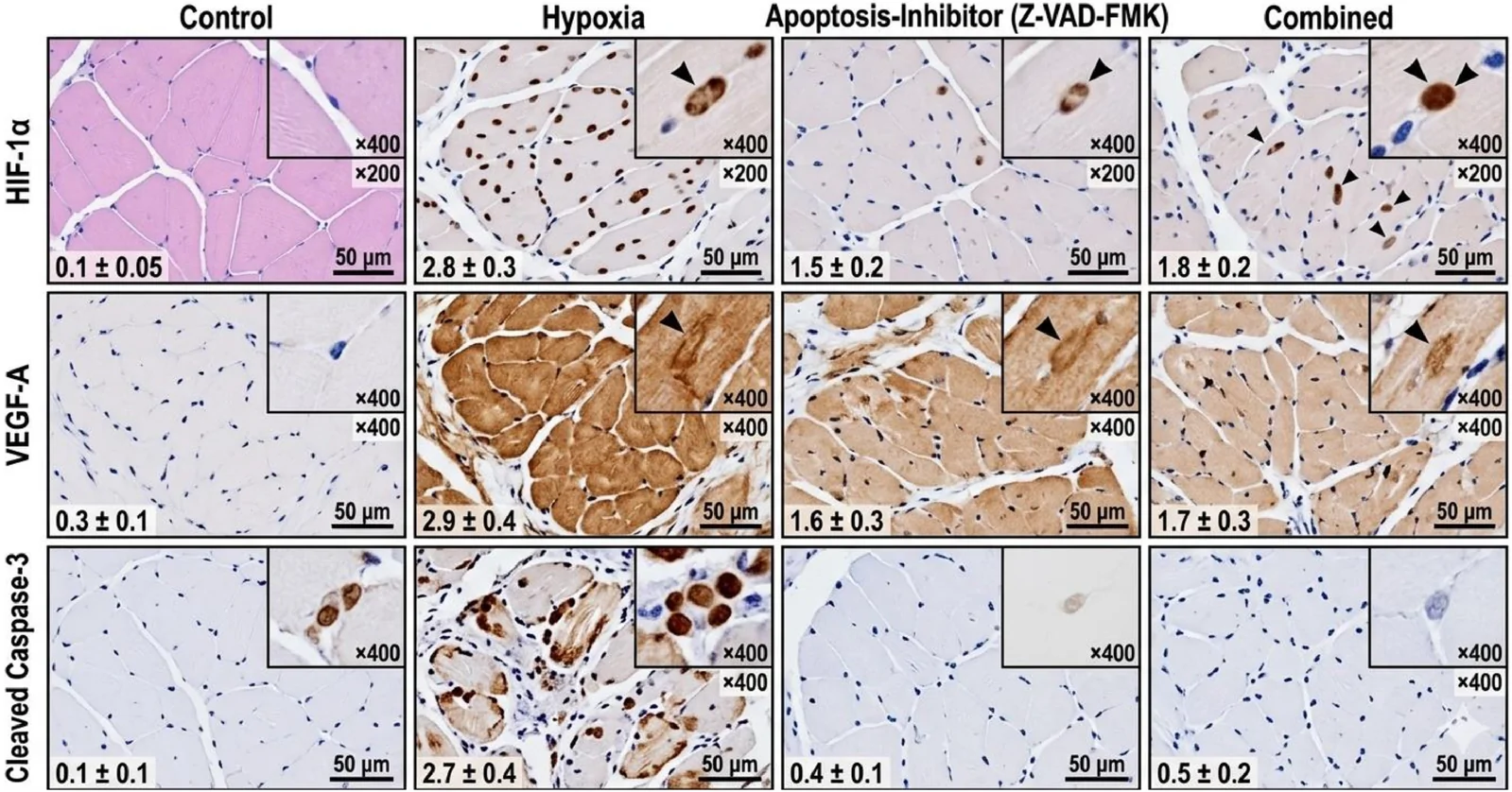
Immunohistochemical localisation of HIF-1α, VEGF-A, and cleaved caspase-3 in pectoralis major muscle at Day 42. Representative immunohistochemistry photomicrographs (× 200) showing nuclear HIF-1α immunoreactivity, cytoplasmic VEGF-A staining, and active (cleaved) caspase-3 (brown DAB chromogen) in the four experimental groups: Control, Hypoxia, Apoptosis-Inhibitor (Z-VAD-FMK), and Combined. Insets show higher-magnification (× 400) detail of positive cells. Semi-quantitative IHC scores (0–3) are presented beneath each panel. Scale bar = 50 µm.

### Apoptosis quantification: TUNEL assay and caspase activity

Results obtained from the TUNEL assay showed an increased apoptotic index (AI) that correlated well with time in the Hypoxia group (Fig. 5A-B). AI was slightly raised in the Hypoxia group by Day 14 (2.1 ± 0.3% vs. 0.4 ± 0.1% in the Control group; p < 0.05). AI rose to 7.8 ± 0.8% in the Hypoxia group vs. 0.6 ± 0.1% in the Control group by Day 28 (p < 0.001). On Day 42, the Hypoxia group showed significantly elevated AI of 14.3 ± 1.2%, whereas the Control group had 0.8 ± 0.1% (p < 0.001). Z-VAD-FMK decreased AI significantly in the Combined group on Day 42 (6.1 ± 0.7%; p < 0.001 vs. Hypoxia).

**Fig. 5:**
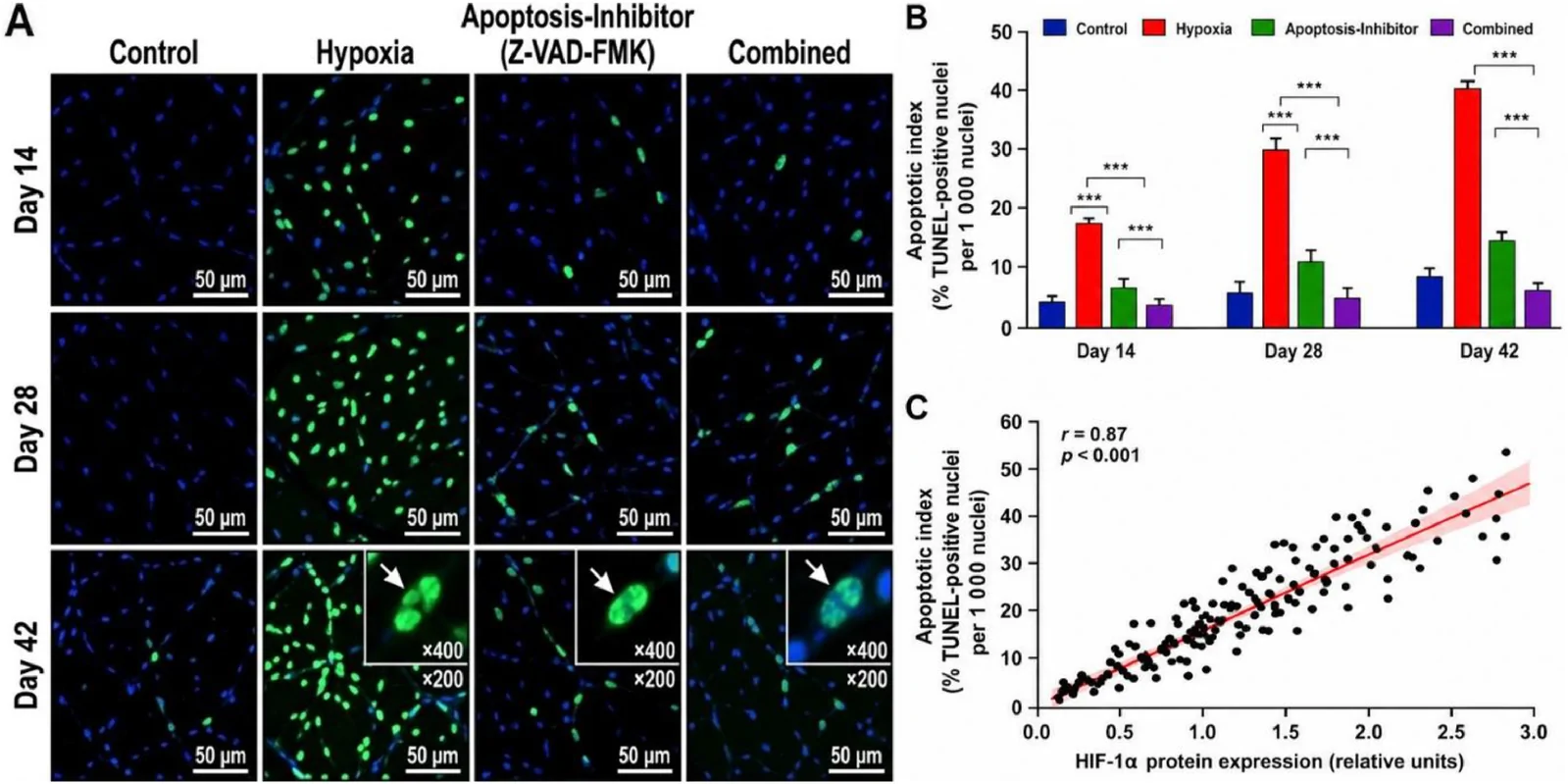
Apoptotic index and TUNEL-positive cell quantification. (A) Representative fluorescent TUNEL-stained sections (green signal, DAPI counterstain in blue) of pectoralis major muscle across the four experimental groups (Control, Hypoxia, Apoptosis-Inhibitor, Combined) at Days 14, 28, and 42 (× 200).(B) Bar graph of apoptotic index (% TUNEL-positive nuclei per 1 000 nuclei counted; mean ± SEM, n = 6 birds, 5 fields/bird). (C) Pearson correlation scatter plot between HIF-1α protein expression (fold-change relative to Day-14 Control) and apoptotic index across individual birds sampled at Days 28 and 42 (n = 72 after listwise deletion of samples missing either measurement). Line of best fit with 95% confidence interval is shown (r = 0.87, 95% CI: 0.80–0.91, p < 0.001).

Pearson correlation analysis performed on individual birds sampled at Days 28 and 42 that possessed complete paired HIF-1α protein and TUNEL data (n = 72 after listwise deletion of incomplete samples) revealed a strong positive correlation between HIF-1α protein expression and apoptotic index (r = 0.87, 95% CI: 0.80–0.91; p < 0.001; Fig. 5C). Caspase-3 enzymatic activity assay confirmed significantly elevated caspase-3 activation in the Hypoxia group at Day 42 (4.2 ± 0.4-fold over Control; p < 0.001; Fig. 6C), which Z-VAD-FMK significantly attenuated in both the Apoptosis-Inhibitor (by 38%; p < 0.001) and Combined (by 51%; p < 0.001) groups.

**Fig. 6:**
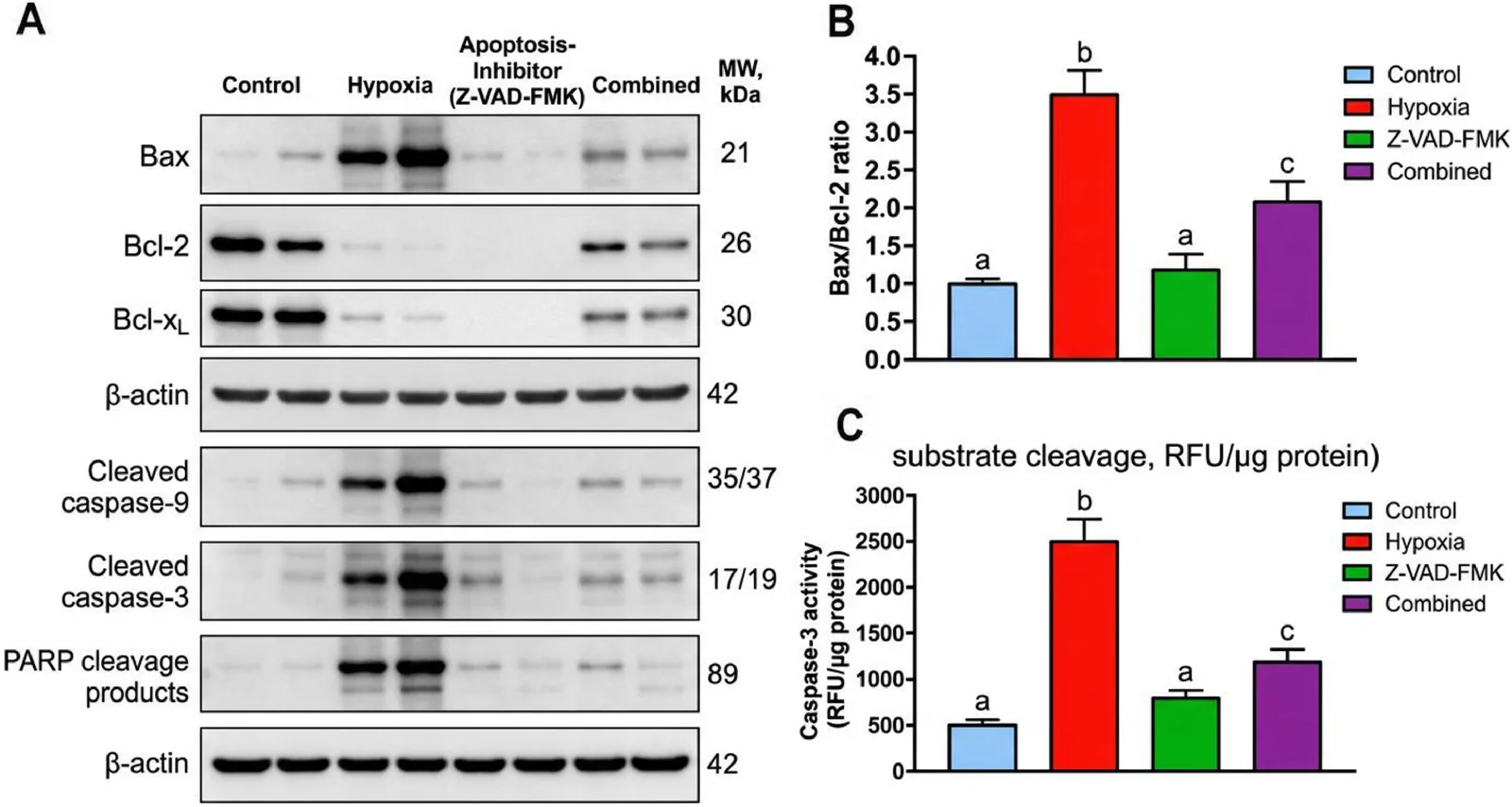
Pro- and Anti-Apoptotic Protein Expression: Bcl-2 Family and Caspase Cascade. (A) Western blot panels for Bax, Bcl-2, Bcl-xL, cleaved Caspase-9, cleaved Caspase-3, and PARP cleavage products at Day 42 across the four experimental groups: Control, Hypoxia, Apoptosis-Inhibitor (Z-VAD-FMK), and Combined. (B) Quantified Bax/Bcl-2 ratio (mean ± SEM). (C) Caspase-3 enzymatic activity (fluorometric substrate cleavage, RFU/µg protein). Z-VAD-FMK–treated birds (Apoptosis-Inhibitor and Combined groups) show significantly attenuated caspase activation. *p < 0.05; **p < 0.01; ***p < 0.001 vs. Hypoxia group.

### Pro- and anti-apoptotic protein expression

Western blot analysis of the Bcl-2 family demonstrated a marked pro-apoptotic shift in the Hypoxia group at Day 42 (Fig. 6A-B). Bax protein was significantly upregulated (3.1 ± 0.3-fold vs. Control; p < 0.001), while anti-apoptotic Bcl-2 was significantly downregulated (0.38 ± 0.05-fold; p < 0.001), yielding a Bax/Bcl-2 ratio of 8.2 ± 0.9 in the Hypoxia group vs. 1.0 ± 0.1 in Controls (p < 0.001). Bcl-xL was similarly downregulated in hypoxic birds (0.42 ± 0.06-fold; p < 0.001). Consistent with Bax/Bcl-2 ratio changes, cleavage of caspase-9 (intrinsic pathway initiator) was significantly elevated in the Hypoxia group (4.7 ± 0.5-fold; p < 0.001), along with downstream cleavage of caspase-3 (4.2 ± 0.4-fold; p < 0.001) and PARP (4.9 ± 0.5-fold; p < 0.001).

In the Combined group, Z-VAD-FMK treatment significantly attenuated cleaved caspase-9 (by 46%), cleaved caspase-3 (by 51%), and PARP cleavage (by 49%) compared with the Hypoxia group (all p < 0.001), without significantly altering the upstream Bax/Bcl-2 ratio (8.0 ± 0.8 in Combined vs. 8.2 ± 0.9 in Hypoxia; p = 0.812).

### Extracellular matrix remodelling and fibrosis

The hydroxyproline level, which is a known biochemical marker for total collagen level, showed a significant increase in the Hypoxia group on Day 42 (7.8 ± 0.6 µg/mg wet tissue compared to 1.9 ± 0.2 µg/mg in Control; p < 0.001; Fig. 7B). The use of Z-VAD-FMK in the Combined group significantly decreased hydroxyproline levels compared to the Hypoxia group (4.8 ± 0.4 µg/mg; p < 0.001), suggesting that the role of apoptosis in the fibrosis process is highly relevant. Quantitative real-time PCR analysis showed significant up-regulation of the *COL1A1* gene (5.2 ± 0.4-fold; p < 0.001) and *COL3A1* gene (4.1 ± 0.3-fold; p < 0.001). TGF-β1 mRNA was co-upregulated (3.8 ± 0.3-fold; p < 0.001) and *TGFB1* protein was elevated in multiplex ELISA (Day 42 Hypoxia: 148.4 ± 12.1 pg/mg vs. 22.3 ± 2.4 pg/mg in Control; p < 0.001), implicating TGF-β1 as a principal fibrotic driver downstream of the hypoxia–apoptosis axis (Fig. 7C).

**Fig. 7:**
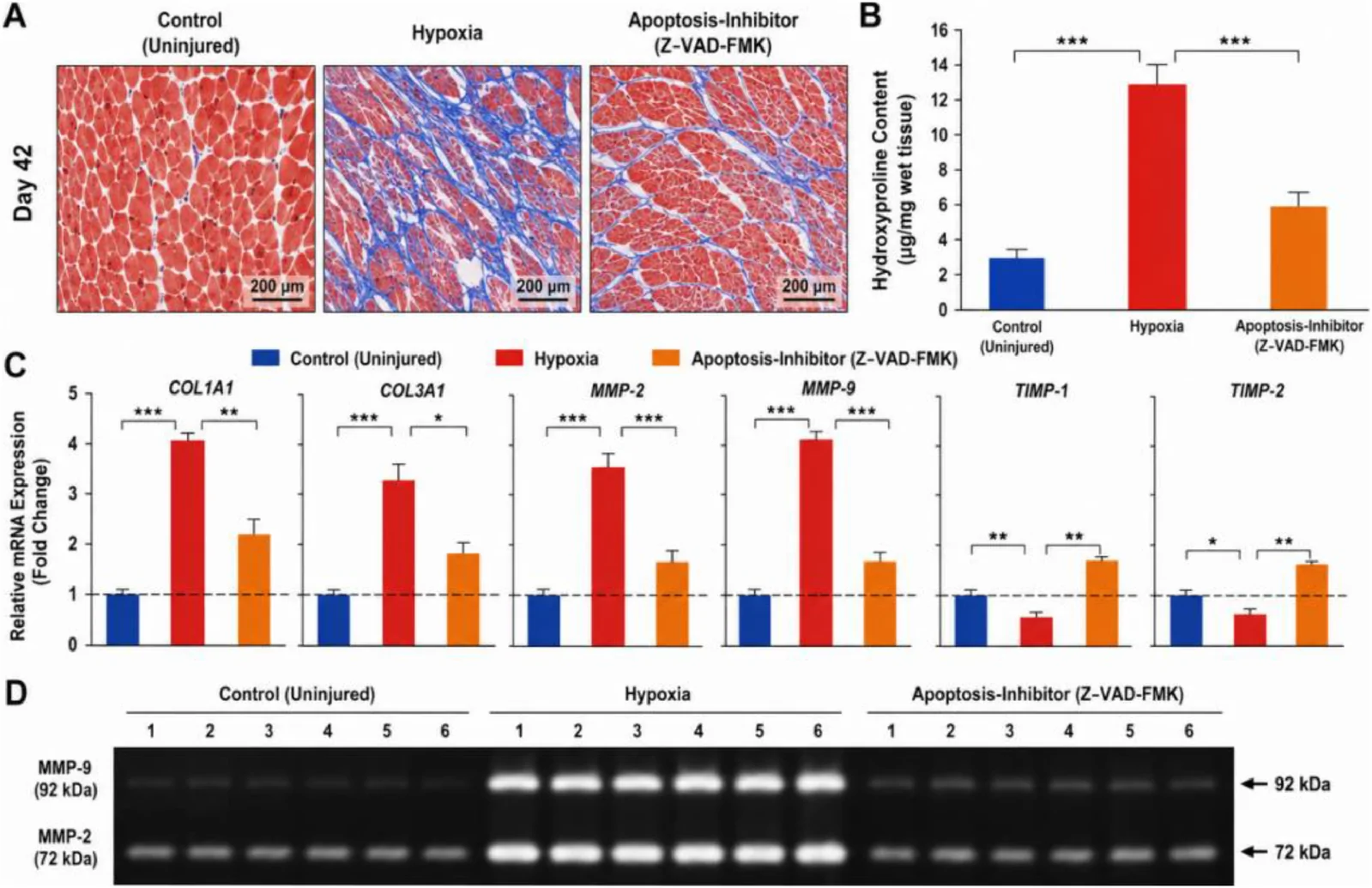
Extracellular Matrix Remodelling: Collagen Deposition and MMP/TIMP Expression. (A) Masson's Trichrome-stained sections showing collagen deposition (blue) in Control, Hypoxia, and Apoptosis-Inhibitor groups at Day 42 (× 100). (B) Hydroxyproline content (µg/mg wet tissue, mean ± SEM). (C) Relative mRNA expression of COL1A1, COL3A1, MMP-2, MMP-9, TIMP-1, and TIMP-2 measured by RT-qPCR. (D) Gelatin zymography showing MMP-2 (72 kDa) and MMP-9 (92 kDa) gelatinolytic activity in muscle extracts.

However, as observed from gelatin zymography, there was a significant elevation in the MMP-2 (72 kDa) and MMP-9 (92 kDa) gelatinase activities in the Hypoxia group on Day 42 (3.4-fold increase in MMP-2 and 4.7-fold increase in MMP-9 activity over control; both p < 0.001; Fig. 7D). Interestingly, despite the upregulation of TIMP-1 and TIMP-2 mRNAs (2.9-fold increase and 2.1-fold increase, respectively; p < 0.001), the ratio between MMP-2 and TIMP-2 and that between MMP-9 and TIMP-1 activities.

### Oxidative stress parameters

Significant oxidative stress was evident in the pectoralis major of hypoxia-exposed birds across all measured parameters (Fig. 8; complete dataset in Supplementary Table S6). At Day 42, the Hypoxia group exhibited markedly elevated MDA levels (1.98 ± 0.14 nmol/mg protein; 4.4-fold over Control; p < 0.001) and protein carbonyl content (1.44 ± 0.12 nmol/mg; 4.5-fold over Control; p < 0.001), indicative of severe lipid peroxidation and protein oxidation. Concurrently, activities of the antioxidant enzymes SOD (6.3 ± 0.7 vs. 18.1 ± 1.4 U/mg in Control; p < 0.001), CAT (14.7 ± 1.5 vs. 40.9 ± 3.2 nmol/min/mg; p < 0.001), and GPx (4.9 ± 0.5 vs. 15.1 ± 1.3 nmol/min/mg; p < 0.001) were severely depleted in the Hypoxia group, as was total GSH (7.8 ± 0.8 vs. 22.1 ± 2.0 nmol/mg; p < 0.001).

**Fig. 8:**
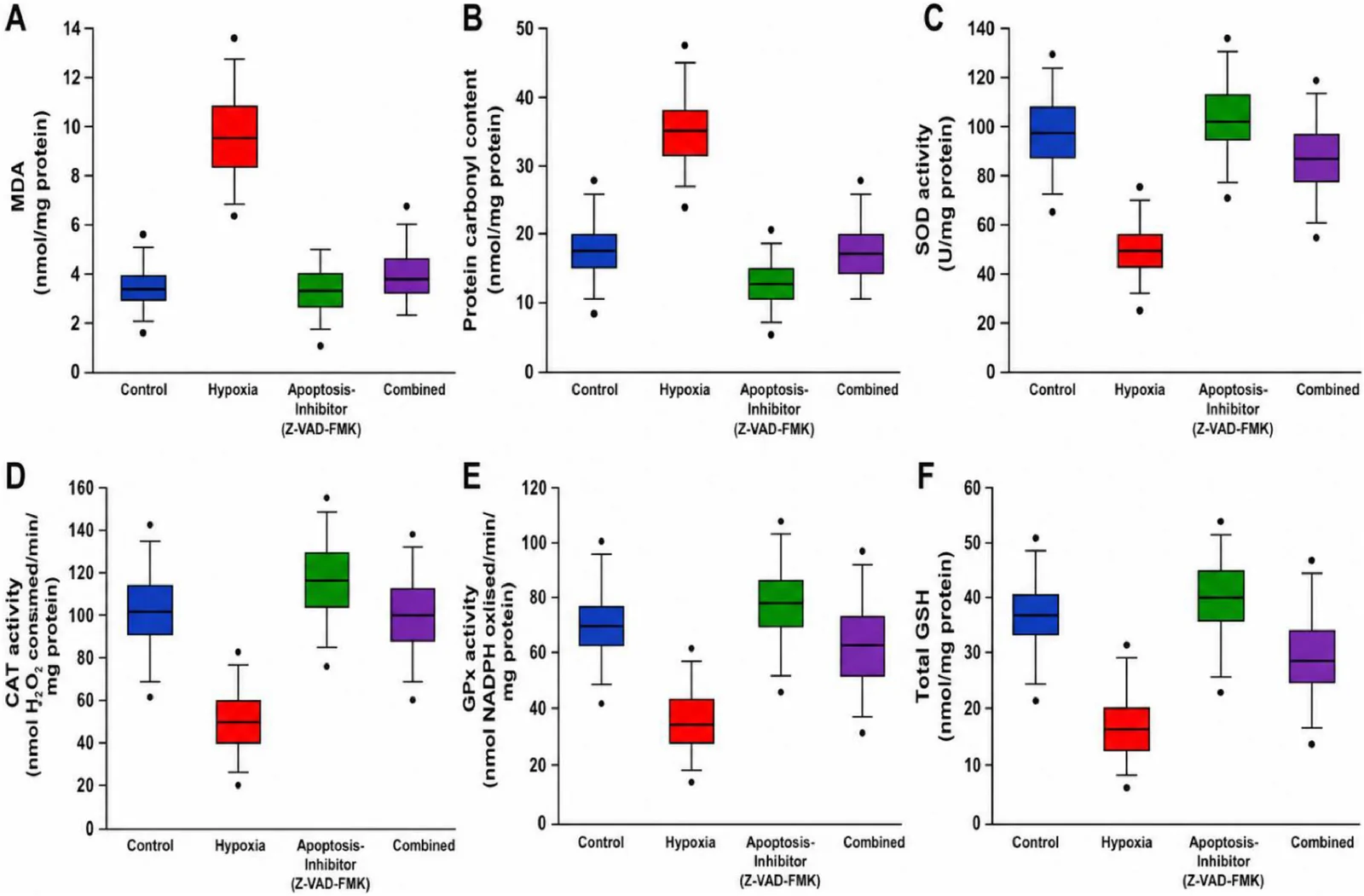
Oxidative Stress and Antioxidant Defence Parameters. Box-and-whisker plots of key oxidative stress biomarkers in pectoralis major muscle tissue at Day 42 across the four experimental groups—Control, Hypoxia, Apoptosis-Inhibitor (Z-VAD-FMK), and Combined: (A) Malondialdehyde (MDA, nmol/mg protein); (B) Protein carbonyl content (nmol/mg protein); (C) Superoxide dismutase (SOD) activity (U/mg protein); (D) Catalase (CAT) activity (nmol H₂O₂ consumed/min/mg protein); (E) Glutathione peroxidase (GPx) activity (nmol NADPH oxidised/min/mg protein); (F) Total glutathione (GSH, nmol/mg protein). Central line = median; boxes = IQR; whiskers = 1.5 × IQR; dots = outliers. n = 12/group.

Z-VAD-FMK treatment in the Combined group partially restored antioxidant enzyme activities and attenuated oxidative damage markers compared with the Hypoxia group at Day 42 (MDA: 0.71 ± 0.07 nmol/mg; SOD: 14.2 ± 1.2 U/mg), suggesting that caspase-mediated cell death contributes to the perpetuation of oxidative stress through release of pro-oxidant cellular contents and disruption of mitochondrial electron transport chain integrity.

### Inflammatory cytokine profile and NF-κB activation

Multiplex ELISA quantification of muscle cytokines revealed a robust pro-inflammatory milieu in hypoxic pectoralis major tissue at Day 42 (Fig. 9A). IL-1β, IL-6, IL-8, and TNF-α were all significantly elevated in the Hypoxia group relative to Controls (IL-1β: 84.3 ± 7.2 vs. 9.1 ± 1.1 pg/mg; IL-6: 112.7 ± 9.8 vs. 12.4 ± 1.4 pg/mg; TNF-α: 76.8 ± 6.4 vs. 8.7 ± 0.9 pg/mg; all p < 0.001). TGF-β1 was substantially elevated (148.4 ± 12.1 pg/mg; p < 0.001), consistent with simultaneous pro-inflammatory and pro-fibrotic signalling.

**Fig. 9:**
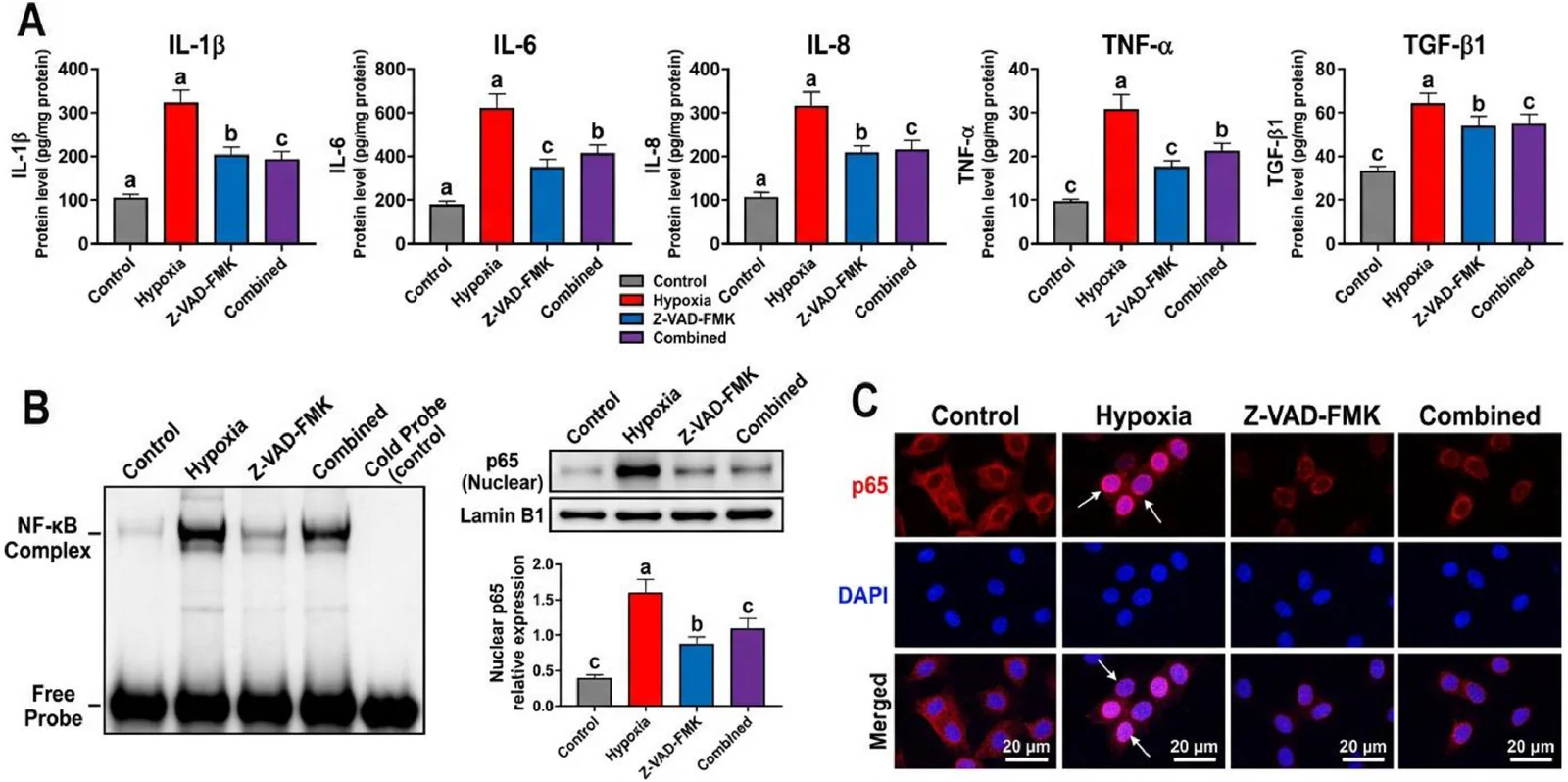
Inflammatory Cytokine Profile and NF-κB Pathway Activation. (A) Multiplex ELISA quantification of IL-1β, IL-6, IL-8, TNF-α, and TGF-β1 protein levels (pg/mg protein, mean ± SEM) in muscle homogenates at Day 42 across the four experimental groups: Control, Hypoxia, Apoptosis-Inhibitor (Z-VAD-FMK), and Combined. (B) NF-κB p65 nuclear translocation assessed by EMSA and Western blot of nuclear fractions from the four experimental groups. (C) Immunofluorescent confocal images showing p65 nuclear localisation (red) and DAPI (blue) in representative birds from Control, Hypoxia, Apoptosis-Inhibitor (Z-VAD-FMK), and Combined groups (× 400). Different letters denote significant differences (Tukey’s HSD, p < 0.05).

Western blot and EMSA analysis of nuclear fractions confirmed significant NF-κB p65 nuclear translocation in the Hypoxia group at Day 42, with nuclear p65 protein elevated 4.1 ± 0.4-fold over Control (p < 0.001; Fig. 9B). Immunofluorescent confocal microscopy corroborated nuclear p65 localisation, showing dense nuclear p65 staining in hypoxic myofibres and interstitial inflammatory cells (Fig. 9C). Z-VAD-FMK treatment in the Combined group attenuated nuclear p65 accumulation by 42% relative to the Hypoxia group (p < 0.001), suggesting that caspase activity amplifies NF-κB-driven inflammatory signalling in hypoxic muscle, potentially through apoptosis-associated release of damage-associated molecular patterns (DAMPs).

### Integrative pathway analysis

The convergent findings across molecular, histopathological, and biochemical analyses support a coherent mechanistic model in which sustained myofibre hypoxia initiates a self-reinforcing cascade comprising HIF-1α stabilisation, mitochondrial dysfunction, oxidative stress, pro-apoptotic Bax/Bcl-2 reprogramming, caspase cascade activation, NF-κB-mediated inflammation, TGF-β1 release, and ECM remodelling culminating in irreversible myofibre loss and fibrotic replacement (Fig. 10). Pharmacological interruption of this cascade at the caspase step (Z-VAD-FMK) significantly attenuated downstream fibrosis, inflammatory signalling, and WB lesion severity without reversing the upstream hypoxic transcriptional programme, identifying executioner caspase activation as a critical amplifying node in WB myopathy pathogenesis. Strong positive correlations were identified between: (i) HIF-1α protein and TUNEL AI (r = 0.87; p < 0.001); (ii) TUNEL AI and hydroxyproline content (r = 0.81; p < 0.001); (iii) Bax/Bcl-2 ratio and MDA concentration (r = 0.78; p < 0.001); and (iv) caspase-3 activity and NF-κB nuclear p65 (r = 0.83; p < 0.001). These correlations collectively support the unifying hypothesis that hypoxia-driven apoptosis is a mechanistic upstream driver rather than an epiphenomenal bystander in the development of fibrotic breast myopathy in fast-growing broiler chickens.

**Fig. 10:**
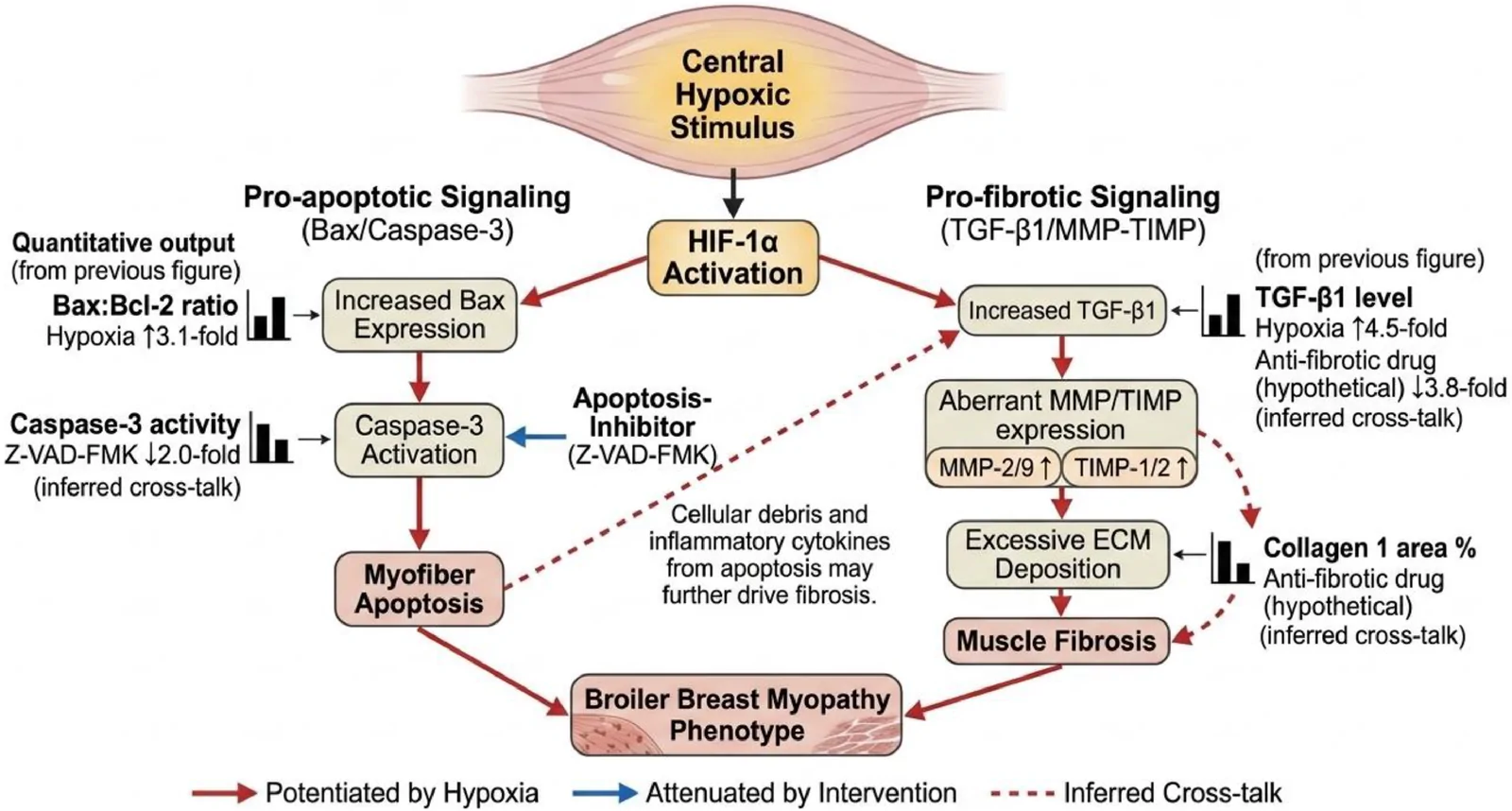
Integrative Pathway Model: Hypoxia–Apoptosis–Fibrosis Axis in Broiler Breast Myopathy. Schematic mechanistic diagram summarising the proposed pathological cascade. Central hypoxic stimulus activates HIF-1α, driving parallel pro-apoptotic (Bax/Caspase-3) and pro-fibrotic (TGF-β1/MMP-TIMP) signalling cascades. Red arrows indicate pathways that are potentiated in the Hypoxia group; blue arrows indicate pathways that are attenuated by Z-VAD-FMK or anti-fibrotic intervention. Dashed lines indicate inferred cross-talk. Key quantitative outputs from the study are annotated at each node.

## Discussion

The present investigation provides, to our knowledge, the first comprehensive characterisation of the temporal sequence and mechanistic interrelationships among hypoxia signalling, apoptotic cell death, oxidative stress, inflammation, and extracellular matrix remodelling in the pectoralis major muscle of broilers with experimentally induced fibrotic breast myopathy. By combining chronic hypobaric hypoxia with pharmacological pan-caspase inhibition across three production timepoints, the study moves beyond correlative observation to establish a causal role for HIF-1α-driven, caspase-dependent apoptosis as an upstream amplifying mechanism in Wooden Breast (WB) pathogenesis. Statistical analysis was structured to directly test the progressive divergence of the hypoxia–apoptosis–fibrosis cascade: two-way ANOVA confirmed significant Group × Time interactions for longitudinal markers (HIF-1α, apoptotic index, caspase-3 activity), while simple main effects at each timepoint provided the direct evidence that hypoxia significantly elevated these markers in a progressive, time-dependent manner. The progressive and marked accumulation of HIF-1α protein in the hypoxic pectoralis major, reaching 4.8-fold over control values by Day 42, is consistent with the well-established mechanism of oxygen-dependent prolyl hydroxylase (PHD)-mediated degradation of HIF-1α under normoxia (Kaelin and Ratcliffe, 2008; Maxwell et al., 1999). In the present model, partial pressure of oxygen equivalent to 13.0% closely replicates the conditions of relative ischaemia proposed to arise within hypertrophied broiler breast muscle masses, where microvascular insufficiency and diffusion-limited oxygen delivery to deep muscle fibres have been documented histologically and by contrast-enhanced imaging (Sihvo et al., 2014, 2018; Papah et al., 2017). The parallel upregulation of *HIF1A* mRNA (3.7-fold) alongside protein accumulation in the Hypoxia group indicates that transcriptional amplification, in addition to post-translational stabilisation, contributes to sustained HIF-1α signalling under chronic hypoxia, a phenomenon also described in chronically hypoxic mammalian skeletal muscle (Semenza, 2012). The concurrent upregulation of *VEGFA* mRNA (2.9-fold) is in keeping with HIF-1α-mediated transcriptional activation of the VEGF promoter (Semenza, 2012) and suggests an attempted but ultimately inadequate angiogenic response, consistent with the progressive nature of WB lesions despite elevated pro-angiogenic signalling. Importantly, Z-VAD-FMK treatment did not significantly alter HIF-1α expression in the Combined group, confirming that the upstream hypoxic transcriptional programme is independent of, and upstream to, the apoptotic cascade modulated by this pharmacological intervention. A pronounced pro-apoptotic shift in the Bcl-2 family protein balance was a hallmark finding in hypoxic pectoralis major muscle, with the Bax/Bcl-2 ratio reaching 8.2 in the Hypoxia group versus 1.0 in controls at Day 42. Bax-mediated mitochondrial outer membrane permeabilisation and consequent cytochrome c release are established triggers of the intrinsic apoptotic cascade (Oltvai et al., 1993), and the downstream sequential activation of caspase-9 and caspase-3 observed here is consistent with engagement of this pathway. These findings align with earlier transcriptomic reports of elevated *BAX* and reduced *BCL2* expression in commercially affected WB muscle (Brothers et al., 2019; Velleman and Clark, 2015), and extend those observations to the protein and enzymatic activity levels with explicit mechanistic interrogation. The strong positive correlation between HIF-1α protein and TUNEL apoptotic index (r = 0.87) across all individual birds supports a mechanistic rather than incidental association, consistent with the documented ability of HIF-1α to transactivate pro-apoptotic BH3-only proteins such as BNIP3 and NIX under prolonged hypoxia (Keith et al., 2012; Semenza, 2012). Critically, pan-caspase inhibition with Z-VAD-FMK in the Combined group reduced the apoptotic index from 14.3% to 6.1% (p < 0.001) and attenuated WB macroscopic lesion scores (median 1.5 vs. 2.5; p < 0.01) and histological lesion area (18.2% vs. 38.4%; p < 0.001) without reversing the upstream Bax/Bcl-2 ratio. This pattern establishes that caspase-dependent execution of cell death, rather than merely mitochondrial dysfunction per se, is a significant effector of structural muscle damage and that partial attenuation of lesion severity is achievable by blocking the execution step of apoptosis. The residual lesion burden in the Combined group likely reflects both the incomplete efficacy of systemic Z-VAD-FMK delivery to deeply hypoxic myofibres and caspase-independent death pathways (e.g., necroptosis, autophagic cell death) that continue to contribute to myofibre loss under sustained hypoxia. The severe depletion of antioxidant defences (SOD, CAT, GPx, total GSH) alongside significant accumulation of oxidative damage markers (MDA, protein carbonyl) in hypoxic pectoralis major muscle at Day 42 corroborates earlier reports of oxidative stress in broiler breast myopathies (; Soglia et al., 2016) and extends them by demonstrating the temporal evolution of oxidative imbalance across the 42-day production period. Reactive oxygen species (ROS) generated during hypoxia-reoxygenation cycles and by dysfunctional mitochondria are well-recognised activators of the intrinsic apoptotic pathway through oxidative modification of Bax and Bcl-2 family proteins and direct cytochrome c release (Valko et al., 2007). The strong positive correlation between the Bax/Bcl-2 ratio and MDA concentration (r = 0.78) observed in the present study supports a bidirectional interaction between oxidative stress and the apoptotic machinery. The partial restoration of antioxidant enzyme activities following Z-VAD-FMK treatment in the Combined group further suggests that caspase-mediated cell death perpetuates oxidative stress through the release of pro-oxidant cellular contents and disruption of mitochondrial electron transport chain integrity, establishing a feedforward cycle between apoptosis and ROS generation that contributes to lesion progression. These findings reinforce the rationale for antioxidant supplementation strategies as adjuncts to genetic or management interventions targeting breast myopathy prevalence in commercial flocks. The robust activation of NF-κB signalling, evidenced by a 4.1-fold increase in nuclear p65 translocation, and the associated marked elevation in pro-inflammatory cytokines (IL-1β, IL-6, IL-8, TNF-α) in hypoxic pectoralis major at Day 42 confirm the presence of a significant inflammatory milieu in WB-affected muscle, as reported in prior histopathological and transcriptomic analyses (Soglia et al., 2016; Tijare et al., 2016). The 42% attenuation of nuclear NF-κB p65 accumulation by Z-VAD-FMK in the Combined group, without direct NF-κB inhibition, suggests that caspase activation amplifies inflammatory NF-κB signalling, plausibly through apoptosis-associated release of danger-associated molecular patterns (DAMPs) such as HMGB1, ATP, and mitochondrial DNA that engage pattern recognition receptors (Soglia et al., 2016). Simultaneous co-upregulation of TGF-β1, a cytokine with both pro-inflammatory and pro-fibrotic properties, to 148.4 pg/mg protein in hypoxic muscle identifies TGF-β1 as a key integrator of the inflammatory and fibrotic arms of WB pathology, consistent with its established role as the principal driver of myofibroblast activation and collagen synthesis in skeletal muscle fibrosis (Velleman and Clark, 2015). The partial attenuation of TGF-β1 and inflammatory cytokines by Z-VAD-FMK in the Combined group further supports the model in which caspase-dependent apoptosis acts as an upstream driver of both inflammatory amplification and fibrotic signalling. The extensive collagen deposition documented in hypoxic pectoralis major muscle, reflected by a 4.1-fold increase in hydroxyproline content and 31.4% fibrotic area on Masson's Trichrome staining at Day 42, represents the histological hallmark of advanced WB myopathy and the primary determinant of consumer-relevant quality defects including altered texture, reduced water-holding capacity, and decreased protein functionality (Bianchi et al., 2018; Petracci et al., 2013). The transcriptional upregulation of *COL1A1* and *COL3A1* (5.2- and 4.1-fold, respectively), in concert with TGF-β1 overexpression, indicates that de novo collagen synthesis by activated myofibroblasts is a major contributor to fibrotic ECM deposition, while the net proteolytic bias towards ECM remodelling demonstrated by elevated MMP-2 and MMP-9 activities despite concomitant TIMP upregulation suggests that MMP-mediated matrix turnover facilitates the substitution of functional contractile tissue with fibrotic stroma. The strong positive correlation between TUNEL apoptotic index and hydroxyproline content (r = 0.81; p < 0.001) across all individual birds implicates apoptotic myofibre death as a proximate trigger for fibrogenic signalling, likely through the release of apoptotic bodies and DAMPs that activate resident fibro-adipogenic progenitor cells and macrophages to adopt a pro-fibrotic phenotype (Velleman and Clark, 2015). The significant reduction in hydroxyproline content (4.8 µg/mg vs. 7.8 µg/mg; p < 0.001) and fibrotic area (19.8% vs. 31.4%; p < 0.001) in the Combined group relative to the Hypoxia group provides the first pharmacological evidence in the broiler model that interruption of caspase-dependent apoptosis substantially limits the downstream fibrotic remodelling response, identifying this pathway as a tractable target for anti-fibrotic intervention. The convergent multi-parameter evidence from this study supports a coherent mechanistic model in which hypoxia-driven HIF-1α accumulation initiates a self-reinforcing pathological cascade characterised by pro-apoptotic Bcl-2 family reprogramming, intrinsic caspase pathway activation, ROS amplification, NF-κB-mediated inflammatory signalling, TGF-β1-driven myofibroblast activation, and MMP-mediated ECM remodelling, collectively culminating in irreversible myofibre loss and fibrotic replacement of pectoralis major tissue. The identification of executioner caspase activation as a critical amplifying node within this cascade has direct translational relevance: it identifies a therapeutic target downstream of the primary hypoxic insult but upstream of the structural fibrotic consequences that are most deleterious to meat quality and animal welfare. While systemic pan-caspase inhibition is not a commercially feasible intervention in commercial broiler flocks, the mechanistic insights provided here support the prioritisation of strategies that reduce myofibre hypoxia as the upstream driver, including improved ventilation and stocking density management, selection against extreme breast muscle hypertrophy, dietary antioxidant supplementation, and targeted genetic approaches to improve intramuscular capillarisation (Petracci et al., 2019; Sihvo et al., 2018). The partial, rather than complete, mitigation of WB severity achieved by Z-VAD-FMK also highlights the existence of caspase-independent death pathways and alternative fibrogenic mechanisms that warrant investigation in future studies. A major limitation of the present study is the use of systemic hypobaric hypoxia (3,000 m; 13.0% O₂) as the experimental insult, which differs fundamentally from the pathophysiology of natural WB in commercial production. The prevailing consensus holds that natural WB arises primarily from extreme pectoralis major hypertrophy outstripping microvascular perfusion capacity, resulting in localized, regional ischaemia/hypoxia within deep muscle fascicles due to diffusion limitation and inadequate capillary density (Sihvo et al., 2014; Papah et al., 2017; Velleman and Clark, 2015). In contrast, our model exposes the entire organism to systemic hypoxaemia, which inevitably produces confounding pathologies including pulmonary hypertension, right-ventricular hypertrophy and failure, and ascites syndrome—the latter documented by the elevated mortality observed between Days 14 and 28. A significant limitation of the present study is the absence of a sham chamber control and the differential housing of normoxic and hypoxic birds. Control and Apoptosis-Inhibitor groups were maintained in floor pens with litter, whereas Hypoxia and Combined groups were housed in hypobaric chambers. Although floor area per bird was matched (0.05 m² per bird), differences in floor type, vertical space, air circulation patterns, and litter availability between pens and chambers introduce potential environmental confounding. An additional confounding factor is the marked difference in body weight between hypoxia-exposed and control birds. By Day 42, Hypoxia birds weighed approximately 16% less than Controls. Because rapid breast-muscle hypertrophy is a well-established risk factor for Wooden Breast, the slower growth trajectory of the hypoxic birds could itself have influenced the severity and progression of the myopathy. It is therefore not possible to fully disentangle the direct effects of reduced oxygen availability from the indirect effects of altered growth rate and muscle development. Notably, Z-VAD-FMK administration partially restored body weight while still significantly reducing apoptotic index, fibrotic area, and inflammatory markers. This observation provides some support for an apoptosis-mediated effect that is at least partly independent of growth rate. No pharmacokinetic data (plasma or tissue concentrations) were generated to verify that therapeutic levels of Z-VAD-FMK were achieved in the pectoralis major. Nevertheless, residual confounding by body weight cannot be excluded, and future studies should consider pair-feeding or weight-matched designs to isolate the specific contribution of hypoxia.Furthermore, final stocking density was higher in normoxic floor pens (∼58 kg/m²) than in hypobaric chambers (∼48 kg/m²) because hypoxia-exposed birds exhibited growth depression; yet WB lesions were far more severe in the hypoxia group, suggesting that oxygen tension remains a dominant factor. Nevertheless, the possibility that chamber-specific stressors (e.g., altered ventilation, absence of litter, or enclosure effects) contributed to the observed pathology cannot be excluded. Future studies should incorporate sham chamber controls (normobaric conditions within identical chamber enclosures) to isolate the specific effect of hypoxia from housing-system artefacts. Table 1, Table 2, Table 3, Table 4, Table 5

**Table 1:** Body Weight, Feed Conversion Ratio, and Wooden Breast Lesion Prevalence Across Experimental Groups and Time Points.

Parameter	Day	Control	Hypoxia	Z-VAD-FMK	Combined	F-statistic	p-value
BW (g)	14	428±21	421±24	430±22	419±25	1.82	0.148
	28	1,612±68	1,389±71^b^	1,598±65	1,421±74^a^	28.4	<0.001
	42	2,891±94	2,418±87^c^	2,847±91	2,634±79^a^	41.3	<0.001
FCR	14	1.42±0.08	1.51±0.09	1.43±0.07	1.54±0.10	2.10	0.107
	28	1.58±0.07	1.84±0.09^b^	1.60±0.08	1.79±0.08^a^	18.7	<0.001
	42	1.71±0.06	2.08±0.10^c^	1.74±0.07	1.93±0.09^b^	31.2	<0.001
WB prevalence (%)	14	0	8.3	0	6.3	—	—
	28	4.2	35.4	6.3	27.1	—	—
	42	6.3	72.9	8.3	41.7	—	—

*Note.* BW = body weight; FCR = feed conversion ratio; WB = Wooden Breast. Values are mean ± SEM (n = 12/group).

a P < 0.05,.

b P < 0.01,.

c P < 0.001 vs. Control at the same time point (Tukey's HSD). FCR was calculated over 14-d intervals.

**Table 2:** Histopathological Quantification: Lesion Area, Fibrotic Area, and Fiber Morphometry at D42.

Parameter	Control	Hypoxia	Z-VAD-FMK	Combined	p-value
Lesion area fraction (%)	1.8 ± 0.3	38.4 ± 3.8^a^	2.1 ± 0.4	18.2 ± 2.6^b^	<0.001
Fibrotic area (% MT-stained)	1.4 ± 0.2	31.4 ± 3.1^a^	1.7 ± 0.3	19.8 ± 2.4^b^	<0.001
Myofiber CSA (µm²)	6,104 ± 418	4,012 ± 296^a^	5,989 ± 401	4,789 ± 341^b^	<0.001
Minimum Feret diameter (µm)	63.2 ± 4.1	47.1 ± 3.3^a^	62.8 ± 3.9	53.8 ± 3.5^b^	<0.001
Type IIb fibers (%)	55.9 ± 3.6	68.4 ± 4.2^a^	56.4 ± 3.8	62.7 ± 4.0*	<0.001
Necrosis score (0-3)	0.1 ± 0.1	2.7 ± 0.2^a^	0.2 ± 0.1	1.6 ± 0.2^b^	<0.001

*Note.* Values are mean ± SEM (n = 12). CSA = cross-sectional area; MT = Masson's Trichrome.

a P < 0.001 vs. Control;.

b P < 0.05 Combined vs. Hypoxia (Tukey's HSD). Lesion and fibrotic area were determined by digital image analysis in QuPath v0.4.3 from a minimum of 5 fields per section.

**Table 3:** HIF-1α Expression, Apoptotic Index, and Caspase-3 Activity Across Groups and Time Points.

Parameter	Day	Control	Hypoxia	Z-VAD-FMK	Combined	Group × Time p	η²p
HIF-1α/β-actin (fold-change)	14	1.00 ± 0.08	1.87 ± 0.18^a^	1.02 ± 0.09	1.91 ± 0.20^a^	<0.001	0.621
	28	1.04 ± 0.09	3.12 ± 0.28^b^	1.06 ± 0.10	3.08 ± 0.31^b^	—	—
	42	1.07 ± 0.10	4.84 ± 0.41^b^	1.09 ± 0.11	4.79 ± 0.44^b^	—	—
TUNEL AI (%)	14	0.4 ± 0.1	2.1 ± 0.3^a^	0.5 ± 0.1	1.9 ± 0.3^a^	<0.001	0.714
	28	0.6 ± 0.1	7.8 ± 0.8^b^	0.7 ± 0.1	5.4 ± 0.6^b^	—	—
	42	0.8 ± 0.1	14.3 ± 1.2^b^	0.9 ± 0.1	6.1 ± 0.7^c^	—	—
Caspase-3 activity (RFU/µg)	14	18.4 ± 1.4	29.2 ± 2.3^a^	11.2 ± 1.1‡	21.4 ± 1.9	<0.001	0.756
	28	19.1 ± 1.6	56.8 ± 4.2^b^	12.4 ± 1.2‡	38.1 ± 3.1^c^	—	—
	42	20.3 ± 1.8	84.7 ± 6.1^b^	12.8 ± 1.3‡	41.6 ± 3.8^c^	—	—

*Note.* AI = apoptotic index; RFU = relative fluorescence units. Values are mean ± SEM (n = 6/group per time point).

a P < 0.05, **P < 0.01,.

b P < 0.001 vs. Control at the same time point;.

c P < 0.001 Combined vs. Hypoxia; ‡P < 0.05 Z-VAD-FMK vs. Control (Tukey's HSD).

**Table 4:** Fibrosis and Extracellular Matrix Remodeling Parameters at D42 Across Experimental Groups.

Parameter	Control	Hypoxia	Z-VAD-FMK	Combined	p-value
Hydroxyproline (µg/mg)	1.9 ± 0.2	7.8 ± 0.6^b^	2.1 ± 0.2	4.8 ± 0.4^c^	<0.001
COL1A1 mRNA (fold-change)	1.00 ± 0.08	5.18 ± 0.41^b^	1.08 ± 0.09	2.94 ± 0.24^c^	<0.001
COL3A1 mRNA (fold-change)	1.00 ± 0.09	4.12 ± 0.34^b^	1.05 ± 0.10	2.61 ± 0.22^c^	<0.001
TGFB1 mRNA (fold-change)	1.00 ± 0.08	3.78 ± 0.31^b^	1.11 ± 0.10	2.18 ± 0.19^c^	<0.001
TGF-β1 protein (pg/mg)	22.3 ± 2.4	148.4 ± 12.1^b^	24.8 ± 2.6	84.7 ± 7.3^c^	<0.001
MMP-2 activity (fold-change)	1.00 ± 0.09	3.41 ± 0.28^b^	1.04 ± 0.11	2.17 ± 0.18^c^	<0.001
MMP-9 activity (fold-change)	1.00 ± 0.10	4.68 ± 0.38^b^	1.07 ± 0.12	2.74 ± 0.23^c^	<0.001
TIMP-1 mRNA (fold-change)	1.00 ± 0.09	2.88 ± 0.24^a^	1.06 ± 0.10	1.94 ± 0.16^c^	<0.001
Fibrotic area, MT (%)	1.4 ± 0.2	31.4 ± 3.1^b^	1.7 ± 0.3	19.8 ± 2.4^c^	<0.001

*Note.* Values are mean ± SEM (n = 12). MMP activity was assessed by densitometric quantification of gelatin zymography bands.

a P < 0.01,.

b P < 0.001 vs. Control;.

c P < 0.05 Combined vs. Hypoxia (Tukey's HSD).

**Table 5:** Cytokine Concentrations and NF-κB Activation in Pectoralis Major Muscle at D42.

Parameter (pg/mg protein)	Control	Hypoxia	Z-VAD-FMK	Combined	p-value
IL-1β	9.1 ± 1.1	84.3 ± 7.2^a^	10.2 ± 1.2	48.6 ± 4.3^b^	<0.001
IL-6	12.4 ± 1.4	112.7 ± 9.8^a^	13.1 ± 1.5	64.8 ± 5.7^b^	<0.001
IL-8	18.3 ± 1.9	98.4 ± 8.6^a^	19.4 ± 2.0	57.2 ± 5.0^b^	<0.001
TNF-α	8.7 ± 0.9	76.8 ± 6.4^a^	9.1 ± 1.0	42.3 ± 3.7^b^	<0.001
TGF-β1	22.3 ± 2.4	148.4 ± 12.1^a^	24.8 ± 2.6	84.7 ± 7.3^b^	<0.001
NF-κB p65 nuclear (fold-change)	1.00 ± 0.09	4.12 ± 0.38^a^	1.08 ± 0.10	2.38 ± 0.22^b^	<0.001

*Note.* Values are mean ± SEM (n = 12). Cytokines were quantified by multiplex bead-based ELISA (LEGENDplex). Nuclear NF-κB p65 was assessed by Western blot of the nuclear fraction, normalized to Lamin B1.

a P < 0.001 vs. Control.

b P < 0.01 Combined vs. Hypoxia (Tukey's HSD).

## Conclusion

This study provides comprehensive mechanistic evidence that chronic myofibre hypoxia drives fibrotic breast myopathy in fast-growing commercial broilers through a sequential, self-amplifying pathological cascade centred on HIF-1α stabilisation, intrinsic caspase-dependent apoptosis, oxidative stress, NF-κB-mediated inflammation, and TGF-β1-driven extracellular matrix remodelling. The progressive accumulation of HIF-1α from Day 14 onward, its strong positive correlation with apoptotic index and fibrosis parameters, and the significant attenuation of WB lesion severity, fibrotic area, and inflammatory signalling by pan-caspase inhibition collectively identify executioner caspase activation as a critical, druggable amplifying node in WB pathogenesis. These findings should be interpreted in light of survivorship bias, as the Day 42 hypoxia cohort represents hypoxia-tolerant survivors and likely underestimates the full pathological severity of chronic hypoxia exposure. These findings establish that hypoxia-driven apoptosis is a mechanistic upstream driver, rather than an epiphenomenal bystander, in the development of fibrotic breast myopathy, and provide a coherent molecular framework to guide the development of targeted interventions—nutritional, genetic, and management-based—aimed at reducing the prevalence and economic impact of this condition in commercial broiler production globally. These conclusions apply specifically to chronic systemic hypoxia-induced myofibrosis and should not be directly equated with the local ischaemic mechanism of natural Wooden Breast in commercial production without further validation.

## Author contributions

Authorship in this manuscript complies with the ICMJE recommendations, under which each author satisfies all four of the following criteria: (1) substantial contributions to the conception or design of the work, or the acquisition, analysis, or interpretation of data; (2) drafting of the manuscript or critical revision of it for important intellectual content; (3) review and explicit approval of the exact final version of the manuscript before publication; and (4) agreement to be personally and publicly accountable for all aspects of the work, including ensuring that any questions regarding the accuracy or integrity of the research are thoroughly investigated and resolved.

**Shixiong Yan** — (i) Substantial contributions: conceived and supervised the overall revision strategy; oversaw the correction of experimental design and parameter records and the verification of all regenerated figures. (ii) Drafting/reviewing: drafted all new limitation paragraphs and performed full-manuscript critical review and verification. (iii) Final approval: reviewed and explicitly approved the exact final version of the manuscript. (iv) Accountability: agrees to be personally and publicly responsible for all aspects of the work and to ensure that any questions regarding the accuracy or integrity of the research are thoroughly investigated and resolved.

**Hongmei Shi** — (i) Substantial contributions: acquisition and analysis of data through re-scoring of Wooden Breast lesions, fibrotic area quantification, immunohistochemistry, and TUNEL counts on archived sections, and verification of the histological data underlying the corresponding figures. (ii) Drafting/reviewing: critically reviewed and revised the histopathology-related portions of the Results and legends for important intellectual content. (iii) Final approval: reviewed and explicitly approved the exact final version of the manuscript. (iv) Accountability: agrees to be personally and publicly responsible for all aspects of the work, including ensuring that questions relating to the accuracy or integrity of the histological data are thoroughly investigated and resolved.

**Muhammad Sarfaraz Iqbal** — (i) Substantial contributions: analysis and interpretation of data through the complete statistical re-analysis, restructuring the analysis to independent one-way ANOVA at each time point, re-deriving the correlation-analysis sample size (n = 72), verifying the Pearson coefficient (r = 0.87), and preparing the supplementary statistical table. (ii) Drafting/reviewing: critically revised the Statistical Analysis section, Results, and Discussion for important intellectual content. (iii) Final approval: reviewed and explicitly approved the exact final version of the manuscript. (iv) Accountability: agrees to be personally and publicly responsible for all aspects of the work, including ensuring that any questions regarding the accuracy or integrity of the statistical reporting are thoroughly investigated and resolved.

**Mikhlid H. Almutairi** — (i) Substantial contributions: acquisition and analysis of data through regeneration of all corrected figure panels and legends, Western blot re-runs and densitometry, and completion of the molecular and pharmacology-related revisions. (ii) Drafting/reviewing: critically revised the molecular-methods and figure-related portions of the manuscript for important intellectual content. (iii) Final approval: reviewed and explicitly approved the exact final version of the manuscript. (iv) Accountability: agrees to be personally and publicly responsible for all aspects of the work, including ensuring that any questions regarding the accuracy or integrity of the molecular data and figures are thoroughly investigated and resolved.

**Gao Yang** — (i) Substantial contributions: conceived the original experimental design, conducted the original animal experimentation, and acquired the primary data that form the foundation of all subsequent work. (ii) Drafting/reviewing: contributed to the original manuscript and critically reviewed the revised manuscript for important intellectual content. (iii) Final approval: reviewed and explicitly approved the exact final version of the manuscript. (iv) Accountability: agrees to be personally and publicly responsible for all aspects of the work, including ensuring that any questions regarding the accuracy or integrity of the original data are thoroughly investigated and resolved.

**Zhang Fei** — (i) Substantial contributions: acquisition of the original data through sample collection and biochemical assays, which form the foundation of all subsequent work. (ii) Drafting/reviewing: contributed to the original manuscript and critically reviewed the revised manuscript for important intellectual content. (iii) Final approval: reviewed and explicitly approved the exact final version of the manuscript. (iv) Accountability: agrees to be personally and publicly responsible for all aspects of the work, including ensuring that any questions regarding the accuracy or integrity of the original biochemical data are thoroughly investigated and resolved.

## Funding

Supported by the Yunnan Fundamental Research Projects (grant NO. 202501AU070088; 202501AU070086), Scientific Research Fund Project of Yunnan Provincial Education Department (Basic Research Special for Young Talents) (grant NO. 2025J0732)

## Disclosures

The authors declare that they have no known competing financial interests or personal relationships that could have appeared to influence the work reported in this paper.

## Data Availability

All data generated or used during this study are included in this published article and its supplementary information files.

## Footnotes

Supplementary material associated with this article can be found, in the online version, at doi:10.1016/j.psj.2026.107805.
