# Supplementary material for: Hypoxia-induced apoptotic pathways and their contribution to fibrosis progression in wooden breast myopathy of fast-growing broilers
Source: Poult Sci. 2026 Sep 21;105(12):107805. doi: 10.1016/j.psj.2026.107805 (PMC13696178; doi:10.1016/j.psj.2026.107805)
Supplement: Supplementary file 1 [file mmc1.docx]

**SUPPLEMENTARY MATERIAL**

**Table S1. Complete Animal Allocation and Housing Conditions by Experimental Group**

| Parameter | Control (n=48) | Hypoxia (n=48) | Apoptosis-Inhibitor (n=48) | Combined (n=48) |
| --- | --- | --- | --- | --- |
| Breed/strain | Ross 308 | Ross 308 | Ross 308 | Ross 308 |
| Sex | Male | Male | Male | Male |
| Initial BW (g) | 43.2 ± 1.8 | 43.5 ± 2.1 | 43.1 ± 1.9 | 43.8 ± 2.0 |
| Housing type | Floor pen (litter) | Hypobaric chamber | Floor pen (litter) | Hypobaric chamber |
| Chambers or pens per group | 4 pens | 4 chambers | 4 pens | 4 chambers |
| Floor area per bird (m²) | 0.05 | 0.05 | 0.05 | 0.05 |
| O₂ level (%) | 20.9 | 13.0 ± 0.3 | 20.9 | 13.0 ± 0.3 |
| Temperature (°C) | 32→22 (wk 1→5) | 32→22 (wk 1→5) | 32→22 (wk 1→5) | 32→22 (wk 1→5) |
| Relative humidity (%) | 50–60 | 50–60 | 50–60 | 50–60 |
| Light program (h) | 23L:1D | 23L:1D | 23L:1D | 23L:1D |
| Feed access | Ad libitum | Ad libitum | Ad libitum | Ad libitum |
| Water access | Ad libitum | Ad libitum | Ad libitum | Ad libitum |
| Z-VAD-FMK dose | Saline vehicle | Saline vehicle | 2 mg/kg i.m. 3×/wk | 2 mg/kg i.m. 3×/wk |
| Final stocking density (kg/m²) | ~58 | ~48 | ~57 | ~53 |
| Mortality rate (%) | 3.1 | 8.3 | 4.2 | 7.1 |

**Table S2. Nutritional Composition of the Basal Diet Used Throughout the Experiment**

| **Nutrient/Ingredient** | **Starter (d 1-14)** | **Grower (d 15-28)** | **Finisher (d 29-42)** |
| --- | --- | --- | --- |
| ME (kcal/kg) | 3,050 | 3,150 | 3,200 |
| Crude protein (%) | 22.5 | 20.0 | 18.5 |
| Lysine (%) | 1.35 | 1.20 | 1.10 |
| Methionine + cystine (%) | 0.97 | 0.88 | 0.82 |
| Calcium (%) | 1.00 | 0.90 | 0.85 |
| Available phosphorus (%) | 0.48 | 0.42 | 0.40 |
| Sodium (%) | 0.18 | 0.18 | 0.18 |
| Vitamin E (IU/kg) | 50 | 50 | 50 |
| Vitamin C (mg/kg) | 200 | 200 | 200 |
| Selenium (mg/kg) | 0.30 | 0.30 | 0.30 |
| Corn (%) | 55.4 | 58.2 | 61.1 |
| Soybean meal (%) | 34.1 | 29.7 | 25.8 |

**Table S3. Complete RT-qPCR Primer Sequences, Amplicon Sizes, and Efficiencies**

| Gene | Forward primer (5′→3′) | Reverse primer (5′→3′) | Amplicon (bp) | Efficiency (%) | GenBank accession |
| --- | --- | --- | --- | --- | --- |
| HIF1A | TGGCGAAGTCATCGAGTTCT | CAGCTTGGATGATGGTGACC | 142 | 98.7 | NM\_204259.1 |
| VEGFA | AGCAAGGTCAAGGTCACGAT | GGCTTTGTTCTGTCTTTCGG | 118 | 97.2 | NM\_205041.1 |
| BAX | GCGTGGTTGCCCTCTTCTAT | CGGTCTGCACAGGGCCTTGA | 135 | 99.1 | NM\_204494.1 |
| BCL2 | CGGATCGTTCCCTGTTTCAT | CAGATCCCGGTTCAGGTACT | 129 | 96.4 | NM\_205339.1 |
| CASP3 | TCAGGCCTGTTGAACTGAAG | ATCGAGTGGGATGTGCACAT | 156 | 98.3 | NM\_204725.1 |
| COL1A1 | GAACCCAAGGAGAAGCTTGT | TGTCCTTTGTCCAGGAATGC | 148 | 97.8 | NM\_204187.1 |
| COL3A1 | CAAAGGTGCAATGAAGGCAG | GCCAGGATCTTGCCAGTCTG | 163 | 96.9 | NM\_001318028.1 |
| MMP2 | CCCACTGCGGTTTTCAACAT | AAGCCAAGGTTGGATGTGAG | 141 | 98.1 | NM\_204420.1 |
| MMP9 | GGCAACGGAGACTTCAACAG | TCTTGTCAGCAGCCACAGTT | 137 | 97.5 | NM\_213781.1 |
| TGFB1 | CGGCATCCTGAACAAGATCC | GGACTGGCGAGCCTTAGTTT | 153 | 98.4 | NM\_204276.1 |
| GAPDH | TCACCAGGGCTGCTTTTAAC | GTTCACACCCATCACAAACAT | 124 | 99.3 | NM\_204305.1 |
| RPL19 | ACCTCCGACTCAGCTACGAA | TGGCAGTACCCTTCCTCTTG | 110 | 99 | NM\_204748.1 |

**Table S4. Antibody Details for Western Blotting and Immunohistochemistry**

| **Target protein** | **Host** | **Clone/cat. no.** | **Supplier** | **WB dilution** | **IHC dilution** | **MW (kDa)** |
| --- | --- | --- | --- | --- | --- | --- |
| HIF-1α | Rabbit | #36169 | Cell Signaling | 1:1000 | 1:200 | 120 |
| VEGF-A | Rabbit | ab46154 | Abcam | 1:500 | 1:100 | 42 |
| Bax | Rabbit | #5023 | Cell Signaling | 1:1000 | 1:200 | 20 |
| Bcl-2 | Mouse | #15071 | Cell Signaling | 1:1000 | 1:200 | 26 |
| Bcl-xL | Rabbit | #2764 | Cell Signaling | 1:1000 | N/A | 26-30 |
| Cleaved caspase-9 | Rabbit | #9505 | Cell Signaling | 1:1000 | N/A | 35/37 |
| Cleaved caspase-3 | Rabbit | #9664 | Cell Signaling | 1:500 | 1:100 | 17/19 |
| PARP (cleaved) | Rabbit | #5625 | Cell Signaling | 1:1000 | N/A | 89 |
| NF-κB p65 | Rabbit | #8242 | Cell Signaling | 1:1000 | 1:200 | 65 |
| MMP-2 | Rabbit | ab37150 | Abcam | 1:500 | 1:100 | 72 |
| MMP-9 | Rabbit | ab38898 | Abcam | 1:500 | 1:100 | 92 |
| β-Actin | Mouse | #3700 | Cell Signaling | 1:5000 | N/A | 42 |

**Table S5. Histopathological Lesion Scoring Criteria for Wooden Breast Myopathy**

| **Score** | **Gross appearance** | **Histological features** | **Fibrosis grade** | **Necrosis (%)** |
| --- | --- | --- | --- | --- |
| 0 (Normal) | Pale pink, soft, normal contour | Normal myofiber morphology, no infiltration | None | 0 |
| 1 (Mild) | Slight pallor, mild firmness | Scattered vacuolar degeneration (<10% fibers) | Minimal (perimysial) | <5 |
| 2 (Moderate) | Diffuse pallor, firm texture | Multifocal necrosis, edema, macrophage infiltration | Moderate (endo+perimysial) | 5-20 |
| 3 (Severe) | White/yellow, very firm, edema | Extensive necrosis, fibroplasia, giant cells, mineralization | Severe (diffuse) | ≥20 |

**Table S6. Complete Oxidative Stress Biomarker Dataset Across All Time Points**

| **Parameter** | **D14 Ctrl** | **D14 Hypox** | **D28 Ctrl** | **D28 Hypox** | **D28 Z-VAD** | **D42 Ctrl** | **D42 Hypox** | **D42 Z-VAD** | **D42 Comb** |
| --- | --- | --- | --- | --- | --- | --- | --- | --- | --- |
| MDA (nmol/mg) | 0.41±0.04 | 0.67±0.07* | 0.43±0.05 | 1.12±0.09** | 0.89±0.08 | 0.45±0.05 | 1.98±0.14*** | 1.02±0.10 | 0.71±0.07 |
| Protein carbonyl (nmol/mg) | 0.28±0.03 | 0.51±0.05* | 0.30±0.04 | 0.87±0.08** | 0.71±0.07 | 0.32±0.04 | 1.44±0.12*** | 0.88±0.09 | 0.62±0.06 |
| SOD (U/mg) | 18.4±1.2 | 13.2±1.0* | 17.9±1.3 | 9.1±0.8** | 12.4±1.1 | 18.1±1.4 | 6.3±0.7*** | 11.8±1.0 | 14.2±1.2 |
| CAT (nmol/min/mg) | 42.1±3.1 | 31.5±2.8* | 41.6±3.4 | 22.3±2.1** | 29.8±2.6 | 40.9±3.2 | 14.7±1.5*** | 26.4±2.3 | 33.1±2.9 |
| GPx (nmol/min/mg) | 15.3±1.1 | 10.8±0.9* | 14.9±1.2 | 7.4±0.7** | 10.1±0.9 | 15.1±1.3 | 4.9±0.5*** | 9.3±0.8 | 11.7±1.0 |
| GSH (nmol/mg) | 22.4±1.8 | 15.6±1.4* | 21.9±1.9 | 11.2±1.0** | 16.4±1.5 | 22.1±2.0 | 7.8±0.8*** | 14.9±1.3 | 18.3±1.6 |

**Table S7. Muscle Fiber Morphometry Data: Cross-Sectional Area and Fiber-Type Distribution**

| **Group** | **Mean CSA (µm²)** | **Minimum Feret (µm)** | **Type I fibers (%)** | **Type IIa fibers (%)** | **Type IIb fibers (%)** | **Fiber density (n/mm²)** |
| --- | --- | --- | --- | --- | --- | --- |
| Control D14 | 2,841±184 | 42.1±2.8 | 18.3±1.6 | 31.4±2.4 | 50.3±3.1 | 248±18 |
| Hypoxia D14 | 2,798±201 | 41.4±3.1 | 17.9±1.7 | 30.1±2.6 | 52.0±3.4 | 251±21 |
| Control D28 | 4,621±312 | 54.8±3.4 | 16.8±1.5 | 29.8±2.3 | 53.4±3.2 | 201±16 |
| Hypoxia D28 | 3,914±284* | 47.2±3.6* | 14.1±1.4 | 26.3±2.1* | 59.6±3.8 | 224±19 |
| Z-VAD D28 | 4,287±297 | 51.6±3.2 | 15.9±1.5 | 28.6±2.3 | 55.5±3.5 | 212±17 |
| Control D42 | 6,104±418 | 63.2±4.1 | 15.4±1.3 | 28.7±2.2 | 55.9±3.6 | 178±14 |
| Hypoxia D42 | 4,012±296*** | 47.1±3.3*** | 10.2±1.1*** | 21.4±1.9*** | 68.4±4.2*** | 239±20* |
| Z-VAD D42 | 5,211±374* | 57.3±3.7 | 13.8±1.2 | 26.1±2.1* | 60.1±3.8 | 196±16 |
| Combined D42 | 4,789±341** | 53.8±3.5* | 12.6±1.2* | 24.7±2.0** | 62.7±4.0** | 208±17 |

**Table S8. Statistical Summary: ANOVA Results and Post Hoc Comparisons for Primary Outcomes**

| **Outcome variable** | **Effect** | **df (num, den)** | **F-statistic** | **p-value** | **η²p** | **Post hoc significance** |
| --- | --- | --- | --- | --- | --- | --- |
| HIF-1α protein | Group | 3, 44 | 48.7 | <0.001 | 0.768 | H vs. C***; H vs. Z*; H vs. Comb** |
| HIF-1α protein | Time | 2, 44 | 82.3 | <0.001 | 0.789 | D42 vs. D14***; D42 vs. D28** |
| HIF-1α protein | Group×Time | 6, 44 | 14.2 | <0.001 | 0.659 | Multiple (see Table 3) |
| Apoptotic index | Group | 3, 44 | 61.4 | <0.001 | 0.807 | H vs. C***; H vs. Z**; H vs. Comb* |
| Bax/Bcl-2 ratio | Group | 3, 44 | 54.9 | <0.001 | 0.789 | H vs. C***; H vs. Z** |
| Caspase-3 activity | Group | 3, 44 | 73.2 | <0.001 | 0.833 | H vs. all*** |
| Hydroxyproline | Group | 3, 44 | 45.8 | <0.001 | 0.758 | H vs. C***; H vs. Z* |
| MDA | Group | 3, 44 | 58.1 | <0.001 | 0.798 | H vs. C***; H vs. Z** |
| TNF-α | Group | 3, 44 | 42.3 | <0.001 | 0.743 | H vs. C***; H vs. Z* |
| IL-6 | Group | 3, 44 | 39.7 | <0.001 | 0.730 | H vs. C***; H vs. Comb* |
| NF-κB p65 (nuclear) | Group | 3, 44 | 36.8 | <0.001 | 0.716 | H vs. C***; H vs. Z* |
| Lesion score (WB) | Group | 3, 188 | 87.4 | <0.001 | 0.582 | H vs. C***; H vs. Z**; H vs. Comb** |
